# Highly branched complex-type N-glycans on integrin β1 in extracellular vesicles enhance the binding of sEVs to laminin on recipient cells

**DOI:** 10.1016/j.jbc.2026.113146

**Published:** 2026-05-14

**Authors:** Tatsuki Isogai, Yuko Tokoro, Miyako Nakano, Koichiro M. Hirosawa, Rinshi S. Kasai, Yasunari Yokota, Yasuhiko Kizuka, Kenichi G.N. Suzuki

**Affiliations:** 1 United Graduate School of Agricultural Science, Gifu University, Gifu, Japan; 2 Institute for Glyco-core Research (iGCORE), Gifu University, Gifu, Japan; 3 Graduate School of Integrated Sciences for Life, Hiroshima University, Higashihiroshima, Japan; 4 Division of Advanced Bioimaging, National Cancer Center Research Institute (NCCRI), Tokyo, Japan; 5 Department of EECE, Faculty of Engineering, Gifu University, Gifu, Japan

**Keywords:** extracellular vesicle, integrin, laminin, microscopy, N-linked glycosylation

## Abstract

Small extracellular vesicles (sEVs) play a crucial role in intercellular communication. Recently, we discovered that sEVs bind to recipient cells *via* interactions between integrin heterodimers and laminins. In this study, we explored how sEV-associated glycans regulate this binding. We performed glycomic analyses of asparagine-linked (N-linked) glycans on integrin β1 in tumor-derived sEVs, revealing that N-glycan structures in sEVs differ from those in their cells of origin. Highly branched, complex-type N-glycans on integrin β1 were predominant in sEVs, whereas less branched complex-type and oligomannose-type N-glycans were more abundant in cells. Furthermore, the sialic acid content of sEV N-glycans was higher than that of cellular glycans. Simultaneous single-particle imaging and super-resolution movie observation revealed that knocking out the glycosyltransferase MGAT5, which synthesizes highly branched complex-type N-glycans, reduced the binding ability of tumor-derived sEVs to laminin on both glass and recipient cell membranes. Although α2,3-sialidase treatment of sEVs did not alter their laminin-binding ability, the α2-3,6,8 sialidase treatment and KO of ST6GAL1 significantly decreased it. Observations following the removal of the N-glycan from the I-like domain of integrin β1 *via* an NQ mutation revealed that the glycan at N269 enhances binding. Additionally, an antibody against the activated state of integrin β1 bound more frequently to WT integrin β1 in sEVs than to the N269Q mutant, indicating that the glycan at N269 is essential for maintaining the protein’s activated conformation. Thus, using advanced imaging techniques, we elucidated the regulatory mechanisms by which N-glycans control the binding of sEVs to laminin on recipient cells.

Extracellular vesicles (EVs) have recently garnered extensive attention as critical mediators of intercellular communication across diverse research fields. EVs, secreted by nearly all cell types, are classified into several categories based on their diameter and cargo molecules, including small EVs (sEVs) and microvesicles. These vesicles encapsulate various cellular components such as proteins, lipids, and genetic materials (including miRNAs), which can be transferred to distant recipient cells, thereby altering their phenotypes (1, 2, 3). Over the past decade, tumor-derived EVs have been recognized for their role in mediating both short-range and long-range crosstalk between tumor and normal cells, facilitating cancer metastasis (4, 5, 6). A previous proteomics study revealed that the composition of integrin heterodimers in sEVs is largely dependent on the cells of origin; furthermore, sEVs containing distinct integrin heterodimers bind to specific recipient cells, potentially influencing organ-selective cancer metastasis *in vivo* (6, 7). However, the molecular mechanisms underlying EV binding to recipient cells remain elusive.

Recently, using high-speed single-molecule imaging and super-resolution microscopy, we demonstrated that α6β1 and α6β4 integrin heterodimers, along with GM1 in tumor-derived sEVs, medium-sized EVs, and microvesicles, exhibit strong binding to laminin on recipient cell plasma membranes (PMs). Conversely, the integrin subunits in these EVs showed minimal binding ability for fibronectin (8). Our results also revealed that the binding of sEVs to laminin on endothelial cells (HUVECs) is essential for inducing cell branching morphogenesis (8). Additionally, our findings indicated that inside-out signaling is not required to sustain the laminin-binding activity of integrins in EVs. In contrast, CD151, a tetraspanin present in EVs, enhances integrin binding to laminin, whereas cholesterol reduces this activity. The membrane composition of EVs differs markedly from that of cellular membranes. For instance, sphingomyelin and gangliosides are enriched in EVs, whereas phosphatidylinositol and phosphatidylcholine are depleted (9), leading to an increased raft-like liquid-ordered phase in EV membranes (10). Membrane proteins in EVs also differ from those in cell PMs; integrins, selectins, and tetraspanins such as CD9, CD81, CD63, and CD151 are enriched in EVs (11, 12). Thus, the binding of EVs to laminin is regulated by the lipids (cholesterol and GM1) and proteins (integrin and CD151) enriched in sEV membranes (8).

It has also been reported that asparagine-linked (N-linked) glycans on integrin subunits play a critical role in regulating integrin function (13, 14). Moreover, N-glycan structures enriched in tumor-derived sEV membranes differ significantly from those in their cells of origin (15, 16). The glycosyltransferase responsible for synthesizing highly branched complex-type N-glycans, MGAT5, is enriched in sEVs derived from cancer cells. After uptake by recipient cells, the endogenous N-glycan structures are remodeled to express MGAT5-produced glycans (17). Furthermore, N-glycans, particularly sialylated forms (18, 19), may regulate the ability of EVs to bind to recipient cells. However, the precise mechanism by which N-glycans regulate EV binding to recipient cells remains unclear. Here, we hypothesized that the integrin N-glycans in sEVs regulate integrin function and control the binding of tumor cell-derived sEVs to recipient cells.

In this study, we clarified the specific structure of N-glycans on integrin β1 in sEVs using glycomic analysis. Based on these results, we assessed the binding ability of tumor-derived sEVs to laminin and recipient cells following glycosyltransferase KO and site-specific removal of glycans from integrin β1. By single-particle tracking and super-resolution movie imaging, we visualized sEVs on living cells at high spatial resolution to investigate whether specific N-glycan structures, particularly those on integrin β1, regulate sEV binding to laminin on the recipient cell PM.

## Results

### N-glycomics of integrin β1 purified from tumor cell-derived sEVs

To characterize the N-glycan structure of integrin β1 in sEVs, we performed an LC-MS glycomic analysis. sEVs were prepared from the culture media of three cancer cell lines (Neuro2A, A549, and B16) using ultracentrifugation, a method previously confirmed to enrich ∼100-nm sEVs, including exosomes (17).

**Figure 1:**
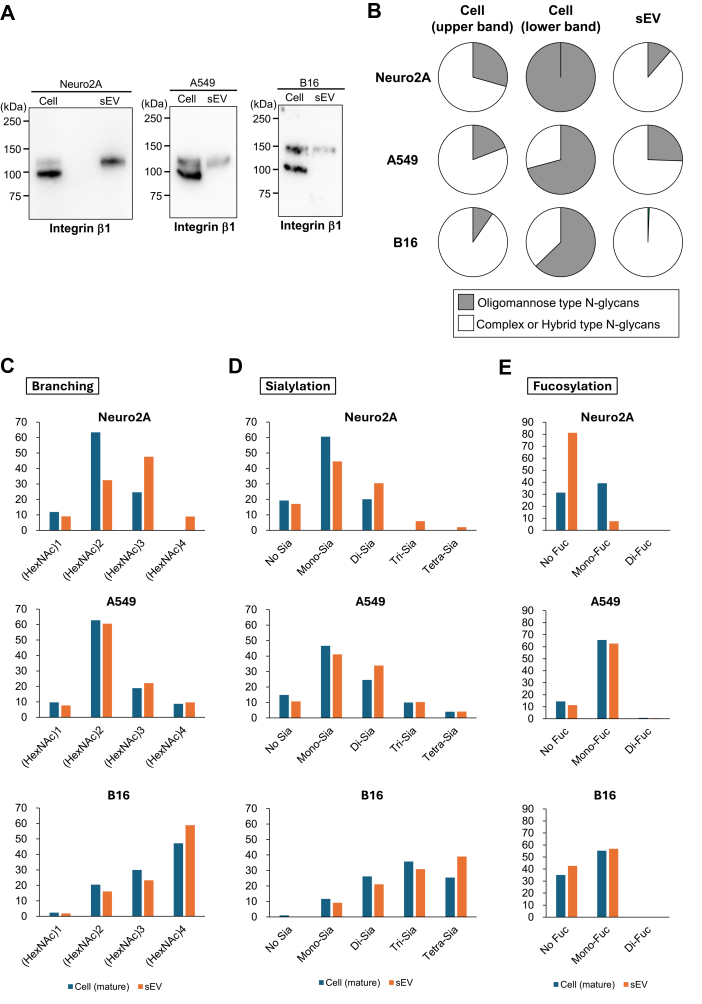
**N-Glycomic analysis of immunopurified integrin β1 derived from Neuro2A, A549, and B16 cells and their sEVs.***A*, Western blotting of immunoprecipitated integrin β1 from Neuro2A, A549, or B16 cells and their secreting sEVs. *B*, N-Glycans were released from 3 forms (*cellular upper band, cellular lower band*, and sEV) of immunoprecipitated integrin β1 and classified. *C*–*E*, Sum of signal intensities of each N-glycan classification in LC-MS analysis is shown. N-Glycans were classified by the number of HexNAc (*C*), Sia (*D*), and Fuc (*E*) residues. For HexNAc, the 2 HexNAc residues in the chitobiose core were not counted. sEV, small extracellular vesicle.

Integrin β1 was immunoprecipitated from both the sEV fraction and parental cells. We observed that cellular integrin β1 yielded two bands, whereas sEV-derived integrin β1 yielded only one band (Fig. 1*A*); this pattern was consistent across all three cell lines. After immunoprecipitation, these three integrin β1 bands were excised from the cell and sEV samples. Subsequently, N-glycans were released using peptide N-glycanase (PNGase F), reduced, and analyzed *via* LC-MS (Fig. 1, *B*–*E* and S1). In total, 110 distinct N-glycans likely attached to integrin β1 derived from either cells or sEVs were detected, with 74, 48, and 53 species identified in Neuro2A, A549, and B16, respectively; overlapping glycans detected in multiple cell types were counted only once in the total (Table S1).

Our analysis revealed that the lower cellular integrin β1 band mainly contained oligomannose-type immature N-glycans, while complex-type N-glycans predominated in the upper cellular band (Fig. 1*B*). This strongly suggests that these two bands represent distinct glycoforms of cellular integrin β1, consistent with previous findings (20). In addition, sEV-derived integrin β1 was primarily decorated with complex type N-glycans (Fig. 1*B*). Classification analysis showed that the number of HexNAc residues in N-glycans of sEV-derived integrin β1 was higher than that in the mature cellular form (upper band), particularly in Neuro2A and B16 cells (Fig. 1*C*). This suggests that the N-glycans on sEV-derived integrin β1 are highly branched. Furthermore, a higher number of sialic acid (Fig. 1*D*) but not fucose (Fig. 1*E*) was detected in N-glycans in sEV-derived integrin β1 than those in cellular integrin β1. These findings suggest that N-glycans of sEV-derived integrin β1 are highly sialylated and branched, compared to those of cellular integrin β1.

### Highly branched N-glycans bearing α2,6-linked sialic acid enhance the binding ability of sEVs to laminin

As demonstrated above, highly sialylated and branched complex-type N-glycans are enriched in cancer cell-derived sEVs. Moreover, earlier studies have suggested that N-glycans and sialic acids on the sEV surface play a role in their uptake by recipient cells and organs (21, 22). However, the precise mechanism through which glycans regulate EV binding remains unclear. Therefore, we aimed to identify the specific glycan structures that regulate EV binding to recipient cells. For subsequent experiments, we focused on sEVs from B16 cells, as their integrin β1 N-glycans were particularly modified with highly branched and sialylated glycans (Fig. 1, *C* and *D*).

To modify N-glycan structures in B16 cells, we knocked out ST6GAL1, a major α2,6-sialyltransferase for N-glycans (23, 24), and MGAT5, which generates the β1,6-GlcNAc branch on the α1,6-Man arm to create highly branched N-glycans (25, 26) (Fig. S2*A*). We confirmed that in ST6GAL1 KO cells, the gene was deleted (Fig. S2, *B* and *C*) and the product α2,6-sialylated glycans were depleted (Fig. S2*D*). We also confirmed the *Mgat5* gene deletion (Fig. S2, *E* and *F*), the loss of MGAT5 protein (Fig. S2*G*), the activity (Fig. S2*H*), and the product glycans (Fig. S2*G*, L4-PHA) in the MGAT5 KO cells.

**Figure 2:**
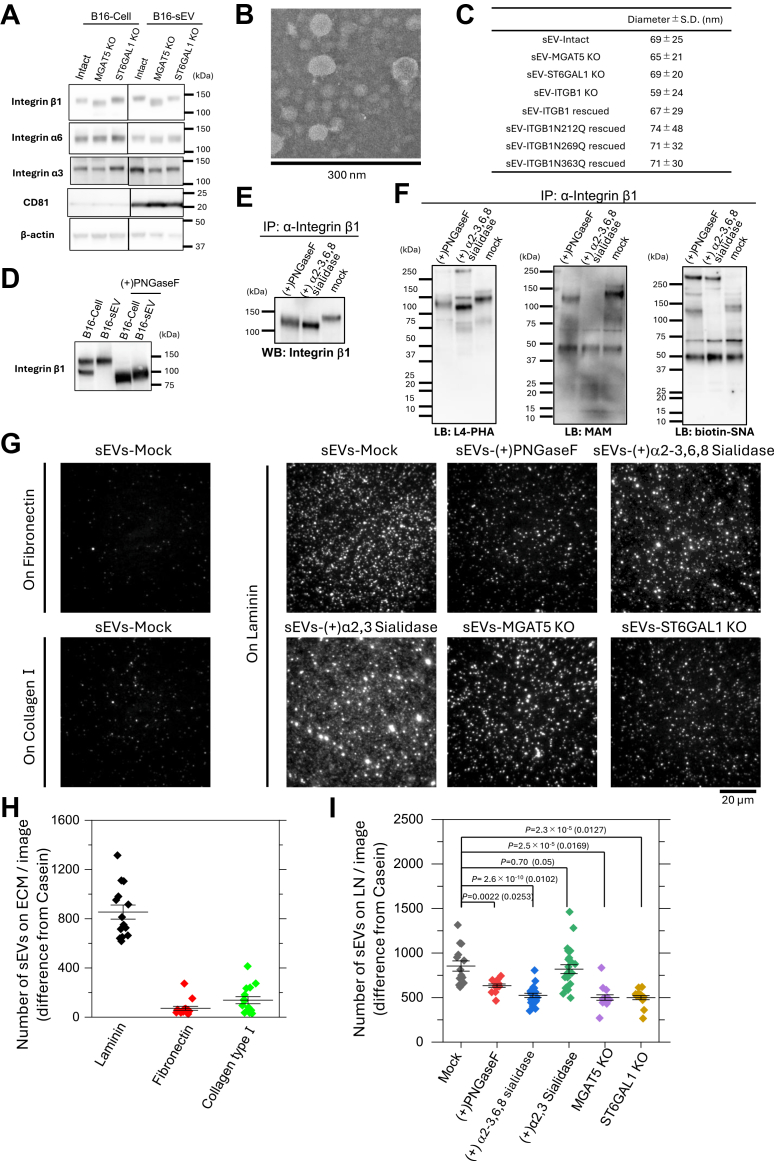
**Single-particle imaging of glycan-engineered B16-derived sEVs attaching to laminin.***A*, Western blot analysis of sEVs derived from intact, MGAT5 KO, and ST6GAL1 KO B16 cells. *B*, Negative-staining transmission electron microscope image of B16 cell-derived sEVs. *C*, The mean sizes of sEVs derived from intact, MGAT5 KO, ST6GAL1 KO, integrin β1 KO, integrin β1-rescued, integrin β1 N212Q-expressed, N269Q-expressed, and N363Q-expressed B16 cell lines. These mean sizes were determined from their transmission electron microscope images. *D*, Western blot analysis of sEVs and cells before and after treatment with PNGase F and α2-3,6,8 sialidase. The molecular weight of PNGase F-treated sEV integrin β1 was lower than that of the intact cellular lower band. In addition, even after PNGase F treatment, the molecular weight of integrin β1 in sEVs remained higher than that of the cellular integrin β1, suggesting that integrin β1 in sEVs undergoes additional post-translational modifications, such as O-glycosylation, to a greater extent than the cellular form. The similar apparent molecular weights observed in the α2-3,6,8 sialidase-treated and PNGase F-treated samples may be attributed to incomplete removal of N-glycans by PNGase F (see *F*) and the presence of sialylated O-glycans on integrin β1. *E* and *F*, Western and lectin blottings of immunoprecipitated integrin β1 from sEVs derived from WT B16 cells after treatment with PNGase F and α2-3,6,8 sialidase, probed with anti-integrin β1 (*E*), L4-PHA, MAM, and SNA (*F*). The mock experiments were performed by incubating sEVs under the same conditions without these enzymes. *G*, Single-particle fluorescence images of sEV-CD81Halo7-SF650T derived from intact, MGAT5 KO, ST6GAL1 KO B16 cells, and those after treatment with PNGase F, α2-3,6,8-sialidase, or α2,3-sialidase on glass coated with laminin, fibronectin, and collagen typeⅠ. *H*, the number of intact B16-sEVs binding to laminin, fibronectin, and collagen typeⅠon glass. *I*, The numbers of the sEVs derived from intact, MGAT5 KO, and ST6GAL1 KO B16 cells, and those after treatment with PNGase F, α2-3,6,8 sialidase, and α2,3-sialidase attached to laminin on glass (*n* = 16 images, independent biological replicates = 3). The data are presented as the mean ± SE. *p*-values were calculated by Welch’s *t* test (two-sided). In *I*, because multiple statistical comparisons were required, the significance levels were corrected using the Holm-Sidak method and are indicated in parentheses. PNGase F, peptide N-glycanase; sEV, small extracellular vesicle.

We focused on examining laminin-binding ability, as we previously discovered that sEVs derived from four different tumor cell lines predominantly bind to laminin on recipient cells (8). In contrast, these sEVs do not bind to fibronectin, despite expressing multiple integrin subunits that function as fibronectin receptors (8). Integrins β1, α6, and α3, components of laminin receptors were expressed in MGAT5 KO and ST6GAL1 KO cells and were present in their sEVs at levels comparable to those inWT sEVs (Fig. 2*A*). Notably, the integrin β1 band in the Western blot shifted to a lower molecular weight following MGAT5 KO, reflecting the altered N-glycan structure. The diameters of the sEVs, as determined by electron microscopy, were not altered by the KO of MGAT5 or ST6GAL1 (Fig. 2, *B* and *C*). Treatment with PNGase F or α2-3,6,8 sialidase decreased the abundance of N-glycans or sialic acids and reduced the molecular weight of integrin β1 on sEVs (Fig. 2, *D* and *E*). This reduction is likely due to the removal of N-glycans and sialic acid on integrin β1. This is supported by the marked reduction in signal intensity observed in lectin blots with L4-PHA, which recognizes β1,6-branched N-glycans (27) after PNGase F treatment, and in those with MAM or SNA, which recognize α2,3-linked (28) or α2,6-linked (29) sialic acids, respectively, after α2-3,6,8 sialidase treatment (Fig. 2*F*).

Subsequently, we performed single-particle imaging of these glycan-remodeled sEVs to quantitatively assess their binding to laminin. Halo7tag-conjugated CD81 was expressed in these KO cell lines, and the tags on their sEVs were fluorescently labeled with SaraFlour650T (SF650T). We selected CD81 as a sEV marker protein because CD81 lacks a consensus sequence for N-glycosylation and is therefore not influenced by glycan alterations. Figure 2*G* shows single-particle images of sEVs bound to laminin, fibronectin, and collagen typeⅠon glass, observed using TIRF microscopy. Because B16-derived sEVs bind preferentially to laminin rather than fibronectin or collagen type I, the binding ability of the glycan-modified sEVs to laminin was investigated further (Fig. 2, *G*–*I*). Quantitative analysis revealed that the number of sEVs bound to laminin was significantly reduced to 74 ± 5.2% (mean ± standard error) of the WT value after PNGase F treatment, suggesting that N-glycans on sEVs enhance their binding ability (Fig. 2*I*). Furthermore, the number of bound sEVs derived from MGAT5 KO and ST6GAL1 KO cells, as well as those observed after sialic acid cleavage by α2-3,6,8 sialidase, were reduced to 59 ± 5.1%, 58 ± 4.6% and 61 ± 4.8%, respectively, relative to those derived from intact cells (Fig. 2*I*). These data indicate that highly branched complex-type N-glycans and α2,6-linked sialic acids enhance sEV binding. The weaker effect of PNGase F compared to ST6Gal1 KO, MGAT5 KO, or α2-3,6,8 sialidase treatment may be attributed to incomplete removal of N-glycans (Fig. 2*F*) and the presence of O-GalNAc glycans on integrin β1. Furthermore, since MGAT5 is enriched on the surface of sEVs and remains functional there (17), it may continue to produce branching of N-glycans in sEVs and thereby attenuate the effects of PNGase F. Conversely, the number of sEVs bound to laminin was not altered by α2,3-sialidase treatment (96 ± 8.4%). While ST6GAL1 adds α2,6- linked sialic acids to terminal galactose residues, α2,3-sialidase specifically cleaves α2,3-linked sialic acids. These results suggest that α2,6-linked sialic acids on galactose, synthesized by ST6Gal1, enhance the binding ability of sEVs to laminin, whereas α2,3-linked sialic acids within sEVs exert minimal influence on this binding.

**Figure 3:**
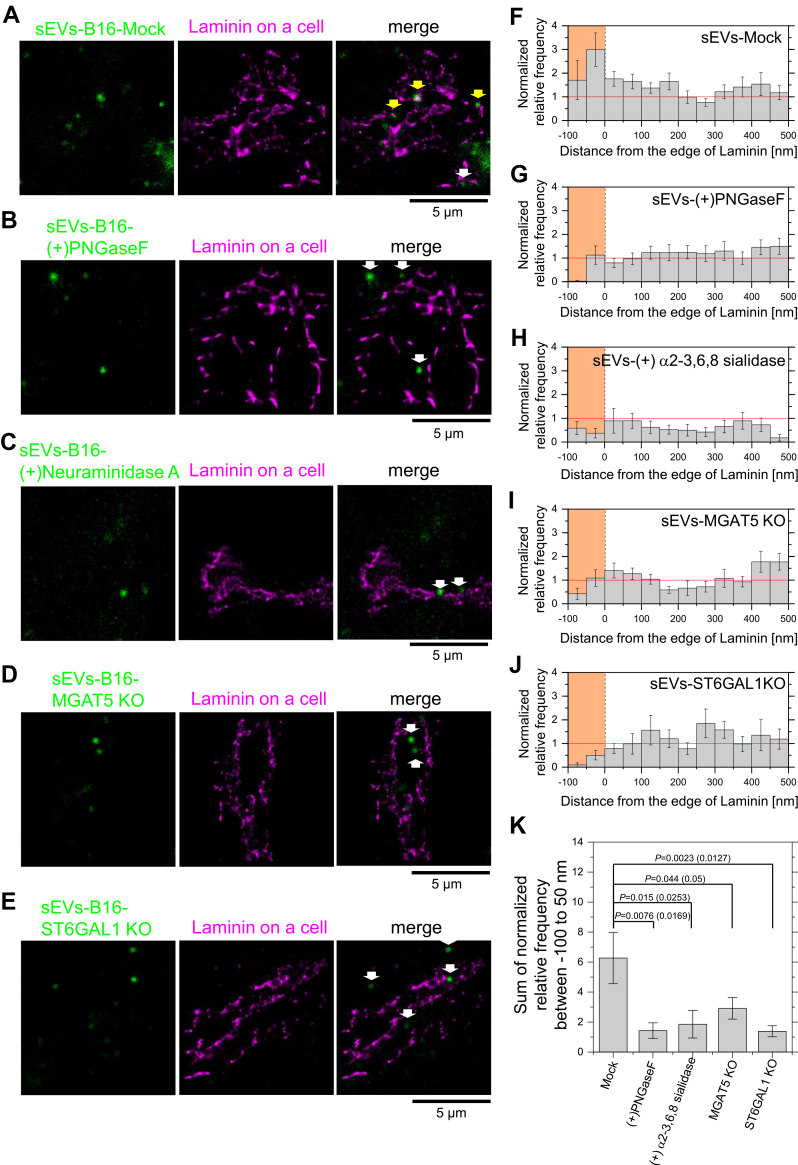
**α2,6-linked sialic acid and highly branched complex-type N-glycans enhance the binding of B16-sEVs to laminin on living cells as revealed by super-resolution microscopy.***A*–*E*, single-particle images of sEV-CD81Halo7-TMR particles (*green*) on an iMEF cell and super-resolution dSTORM images of laminin (magenta) on the iMEF cell after 30 min of incubation. sEVs derived from intact (*A*), MGAT5 KO (*D*), and ST6GAL1 KO (*E*) B16 cells or those after treatment with PNGaseF (*B*) and α2-3,6,8 sialidase (*C*) were observed. sEVs localized near the boundary of laminin structures and sEVs localized alone are indicated by *yellow* and white arrows, respectively. *F*–*J* probability density analysis of each glycan-engineered sEVs-CD81Halo7-TMR and laminin structures on the iMEF cell (*n* = 20 cells, independent biological replicates = 3). The distances from the laminin structures to the sEVs were measured, and the sEV localizations were represented as a histogram of these distances. The normalized relative frequency was defined as the ratio of these sEV distributions to the distribution of computer-generated random spots. A ratio greater than 1 indicates enrichment of sEVs at a given location. When sEVs were localized on the contour of the laminin structure in the dSTORM images, as determined by the kernel density estimation method, their distances were defined as zero; when they were localized within the laminin structure, and their distances were assigned negative values. *K*, the sum of normalized relative frequency between −100 nm and 50 nm as a colocalization index. *p*-value was calculated by Welch’s *t* test (two-sided). In *K*, because multiple statistical comparisons were required, the significance levels were corrected using the Holm-Sidak method and are indicated in parentheses. sEV, small extracellular vesicle.

Next, to investigate the effect of glycan modifications on the sEV binding to laminin on the PM of living cells, we simultaneously performed super-resolution dSTORM observation of laminin and single-particle tracking of sEVs derived from intact (Fig. 3*A* and Movie S1), PNGase F-treated (Fig. 3*B*), α2-3,6,8 sialidase-treated (Fig. 3*C*), MGAT5 KO (Fig. 3*D* and Movie S2), and ST6GAL1 KO (Fig. 3*E* and Movie S3) B16 cells on the living cell PM. Since both sEVs and laminin move slowly on the PM, altering their positions during observation, we aimed to more precisely analyze the colocalization between sEVs and laminin by acquiring super-resolution “dSTORM movies” instead of still images. To quantitatively assess this colocalization, we measured the nearest distance from the edge of the laminin structure to the centroid of the sEV spot for all pairs, calculating the number density of sEVs at each distance. In this analysis, localization to laminin structures is represented by a negative value. The data were normalized using the *in silico* number density of randomized sEV spots. Subsequently, we obtained histograms showing the distribution of normalized relative frequencies of sEVs at each distance from the edge of the laminin structures (Fig. 3, *F*–*J*) (8, 30). To evaluate colocalization, the sum of normalized relative frequencies between −100 nm and 50 nm was determined. Because the diameter of the isolated sEVs is approximately 70 nm (Fig. 2*C*), the center of a particle attached laterally would be positioned ∼40 nm from the laminin surface; thus, the threshold was set to 50 nm. After treatment with PNGase F, MGAT5 KO and ST6GAL1 KO, the degree of colocalization was reduced to 31 ± 10% (mean ± S.E.), 46 ± 16%, and 22 ± 8%, respectively, relative to sEVs from intact cells (Fig. 3*K*). These results demonstrate that highly branched complex-type N-glycans and α2,6-linked sialic acid on N-glycans enhance sEV binding to laminin on the cell membrane, consistent with the results observed on glass (Fig. 2*I*).

### Glycan at N269 of integrin β1 enhances the binding ability of sEVs to laminin

A previous study suggested that integrins α6β1 and α6β4 in sEVs from 4175-LuT human breast cancer cells determine the selectivity of lung metastasis (7). Our previous findings also demonstrated that integrins α6β1 and α6β4 are implicated in the binding of sEVs derived from human PC3 (prostate cancer) cells to laminin (8). Moreover, it has been reported that the functions of integrin subunits are regulated by N-glycans attached to these subunits (31). Consequently, we hypothesized that N-glycans on integrin subunits modulate integrin function in sEVs derived from B16 melanoma cells. Since our results showed that KO of integrin β1 in PC3 cell-derived sEVs reduced their binding ability to laminin by 75%—the most significant among integrin subunits (8)—we focused on investigating the role of glycans of integrin β1 function. Previous studies have shown that the removal of glycans at N212, N269, and N363 in the I-like domain of the integrin β1 subunit decreases both the expression levels and α5β1 heterodimeric formation in cell PMs, which in turn inhibits cell spreading (13, 14). Furthermore, structural analysis revealed that this domain forms binding interfaces between integrin and laminin and between integrin αβ subunits (32), raising the possibility that the N-glycans at these sites could play vital roles in integrin-laminin interaction and/or heterodimer formation of integrin.

**Figure 4:**
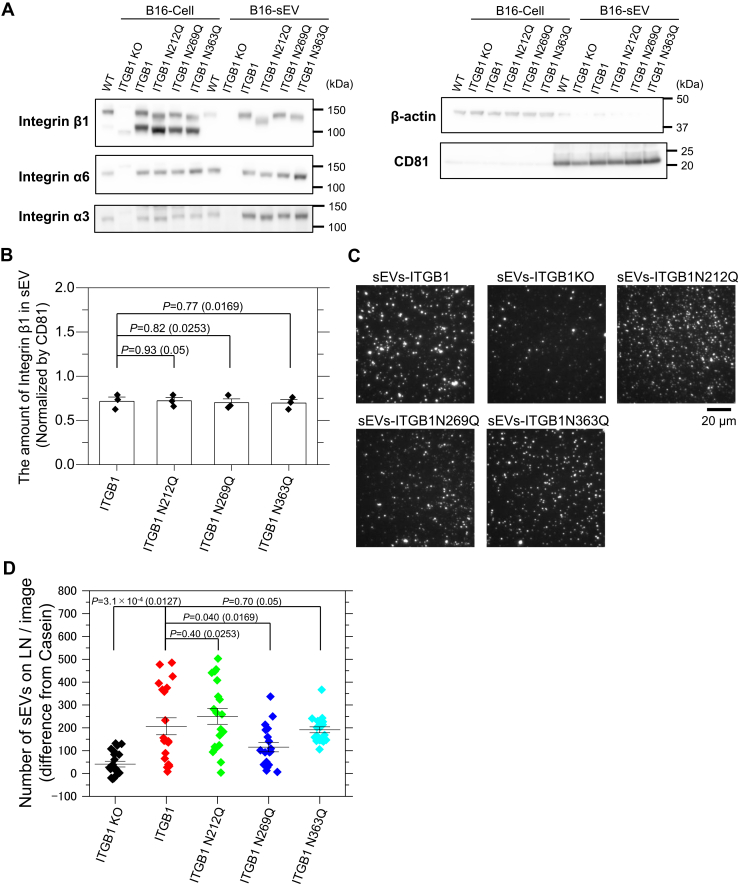
**Glycan of integrin β1 at N269 in sEVs enhances their binding affinity to laminin on glass or the number of activated integrin β1.***A*, Western blotting analysis of WT B16 cells, integrin β1-KO cells, integrin β1-rescued, integrin β1 N212Q, N269Q, and N363Q mutant-expressed cells, and sEVs derived from those cell lines. *B*, quantitative analysis of the amounts of integrin β1 in integrin β1-rescued, integrin β1 N212Q, N269Q, and N363Q mutant-expressed sEVs by western blotting. *C*, single-particle fluorescence images of sEV-CD81Halo7-SF650T on glass coated with laminin. *D*, The numbers of the sEVs attached to laminin on glass (*n* = 20 images, independent biological replicates = 3). The data are presented as the mean ± SE. *p*-value was calculated by Welch’s *t* test (two-sided). In *B* and *D*, because multiple statistical comparisons were required, the significance levels were corrected using the Holm-Sidak method and are indicated in parentheses.

To investigate the function of these N-glycans, we generated integrin β1-KO B16 melanoma cells and stably expressed integrin β1 WT, N212Q, N269Q, or N363Q mutants in KO cells. Gene deletion in the KO cells was confirmed through genotyping PCR (Fig. S3*A*), genomic sequencing (Fig. S3*B*), and western blotting (Fig. S3*C*); all the mutants were expressed at levels comparable to those of the WT (Fig. S3*D*). Integrin β1 WT, N212Q, N269Q, or N363Q mutants were equally incorporated into the sEVs derived from the cell lines (Fig. 4, *A* and *B*). Western blotting revealed that integrin β1 KO reduced the expressions of integrins α6 and α3 (Fig. 4*A*). However, exogenous expression of either integrin β1 WT or NQ mutant restored the expression of integrin α6 and α3 (Fig. 4*A*). The expression levels of integrin β1 WT, N212Q, N269Q, and N363Q mutants in sEVs were comparable to each other (Fig. 4, *A* and *B*), and the levels of integrin α6 and α3 in sEVs expressing the β1 N212Q, N269Q, and N363Q mutants were similar to those of integrin β1-rescued sEVs (Fig. 4*A*).

**Figure 5:**
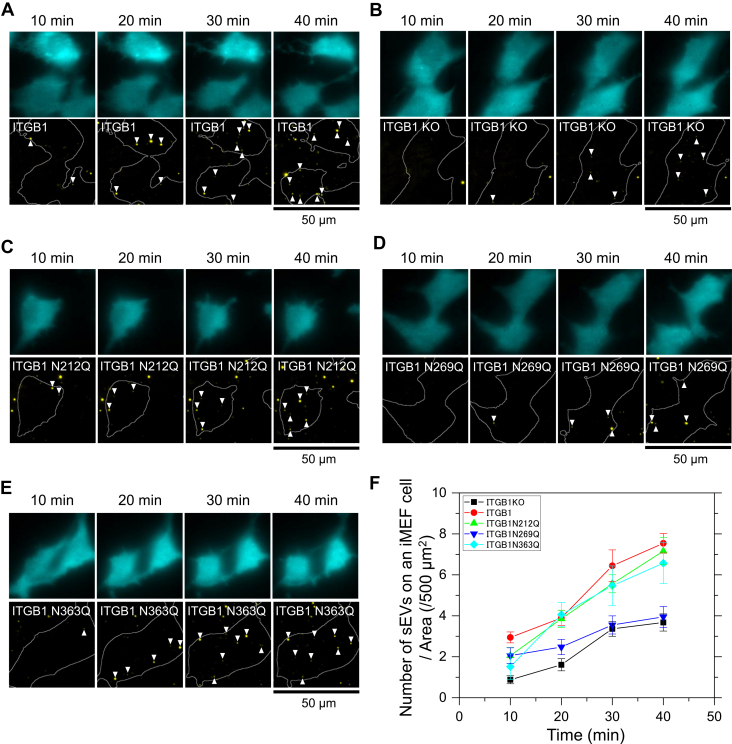
**Single-particle imaging of sEVs containing integrin β1 NQ mutant attached to living iMEF cells.***A*–*E*, single-particle images of B16 cell-derived sEVs containing CD81Halo7-SF650T (*yellow*) on iMEF cells expressing GFP (*cyan*) after 10, 20, 30, 40 min of incubation with these sEVs (*A*). Integrin β1-KO cell-derived sEV (*B*), or sEVs derived from cells expressing N212Q (*C*), N269Q (*D*), and N363Q mutants of integrin β1 (*E*), were also observed as well and the numbers of sEVs bound to cells were analyzed. Lines in the bottom images indicate the edge of binary iMEF-GFP images, and sEVs on the iMEF cell membrane are shown by white arrowheads. *F*, time course of the number of sEVs derived from integrin β1-KO, intact integrin β1-rescued, integrin β1 N212Q, N269Q, N363Q mutant-expressed B16 cells that were bound to the iMEF cell membrane (*n* = 12 cells, independent biological replicates = 1). The data are presented as the mean ± SE. sEV, small extracellular vesicle.

To evaluate the ability of sEVs containing the integrin β1 mutants to bind to laminin, we performed single-particle imaging of sEVs on laminin-coated glass (Fig. 4*C*). The number of sEVs containing the N269Q mutant bound to laminin on glass tended to be lower than that of sEVs containing integrin β1 WT, with a reduction of 56 ± 14% (Fig. 4*D*). In contrast, the number of sEVs containing other mutants bound to laminin was comparable to that containing integrin β1 WT (Fig. 4*D*). These findings suggest that the glycan at N269 of integrin β1 enhances the binding affinity of integrin β1 to laminin on glass or the number of activated integrin β1. Additionally, we examined the time course of the number of sEVs containing integrin β1 WT or its NQ mutants bound to recipient cells (Fig. 5, *A*–*E*). The quantitative analysis explicitly indicated that after the removal of the glycan at N269 of integrin β1, the number of bound sEVs after 40 min of incubation was significantly reduced to 52 ± 8% of those containing integrin β1 WT (*p* = 1.8 × 10^−5^) (Fig. 5*F*).

**Figure 6:**
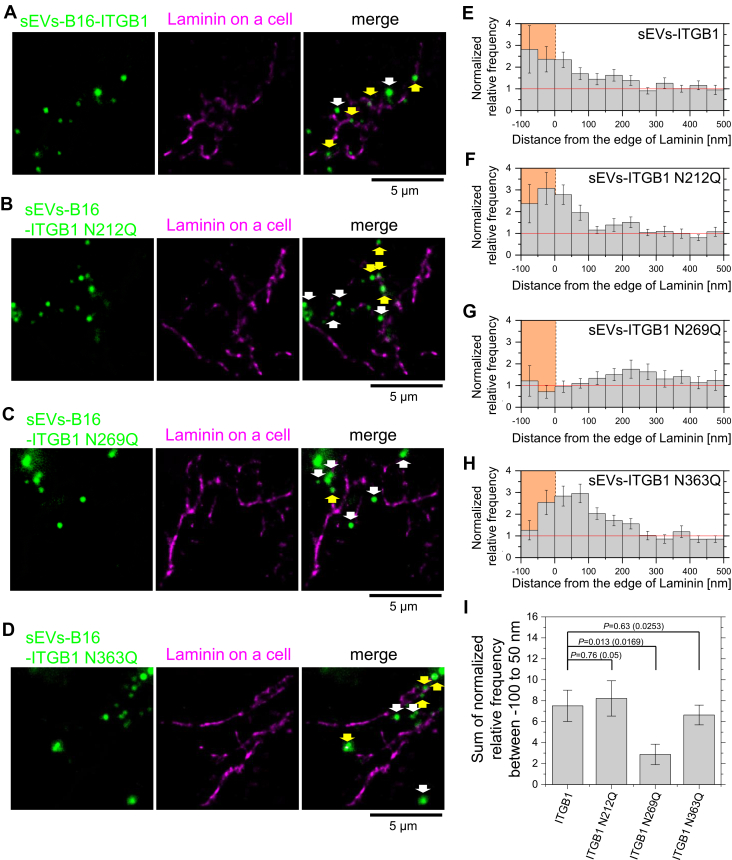
**Glycan at N269 of integrin β1 enhances the binding ability of sEVs to laminin on living iMEF cells.***A*–*D*, simultaneous observation of single-particles of sEVs-CD81Halo7-TMR derived from intact integrin β1-rescued (*A*) or integrin β1 N212Q (*B*), N269Q (*C*) and N363Q (*D*) mutant-expressed B16 cells (*green*) and super-resolution dSTORM image of laminin (*magenta*) on the iMEF cell after 30 min of incubation. sEV particles localized near the boundary of laminin structures and sEV particles localized alone are indicated by yellow and white arrows, respectively. *E*–*H*, probability density analysis of sEV particles and laminin structures on iMEF cell membrane (*n* = 24 cells, independent biological replicates = 3). The normalized relative frequency was defined as the ratio of the number densities of these sEVs at each distance from laminin structures to that of randomly distributed spots generated by a computer. *I*, the sums of normalized relative frequency between −100 nm and 50 nm. *p*-value was calculated by Welch’s *t* test (two-sided). In *I*, because multiple statistical comparisons were required, the significance levels were corrected using the Holm-Sidak method and are indicated in parentheses. sEV, small extracellular vesicle.

Next, we investigated whether the ability of sEVs to bind to laminin on a living cell was reduced after the removal of N-glycan. We simultaneously performed super-resolution dSTORM observations of laminin and single-particle tracking of sEVs derived from integrin β1 WT-rescued (Fig. 6*A* and Movie S4), N212Q (Fig. 6*B*), N269Q (Fig. 6*C* and Movie S5), and N363Q mutant-expressed (Fig. 6*D*) B16 cells. The cumulative normalized relative frequency of sEVs containing the integrin β1 N269Q mutant within the range of −100 to 50 nm was 2.9 ± 1.0, which is markedly lower than that of sEVs containing integrin WT or other NQ mutants (Fig. 6, *E*–*I*). These findings suggest that the glycan on N269 of integrin β1 enhances its ability to bind to laminin on the cell PM.

### Glycan at N269 of integrin β1 maintains the active conformation of integrin dimers in sEVs

**Figure 7:**
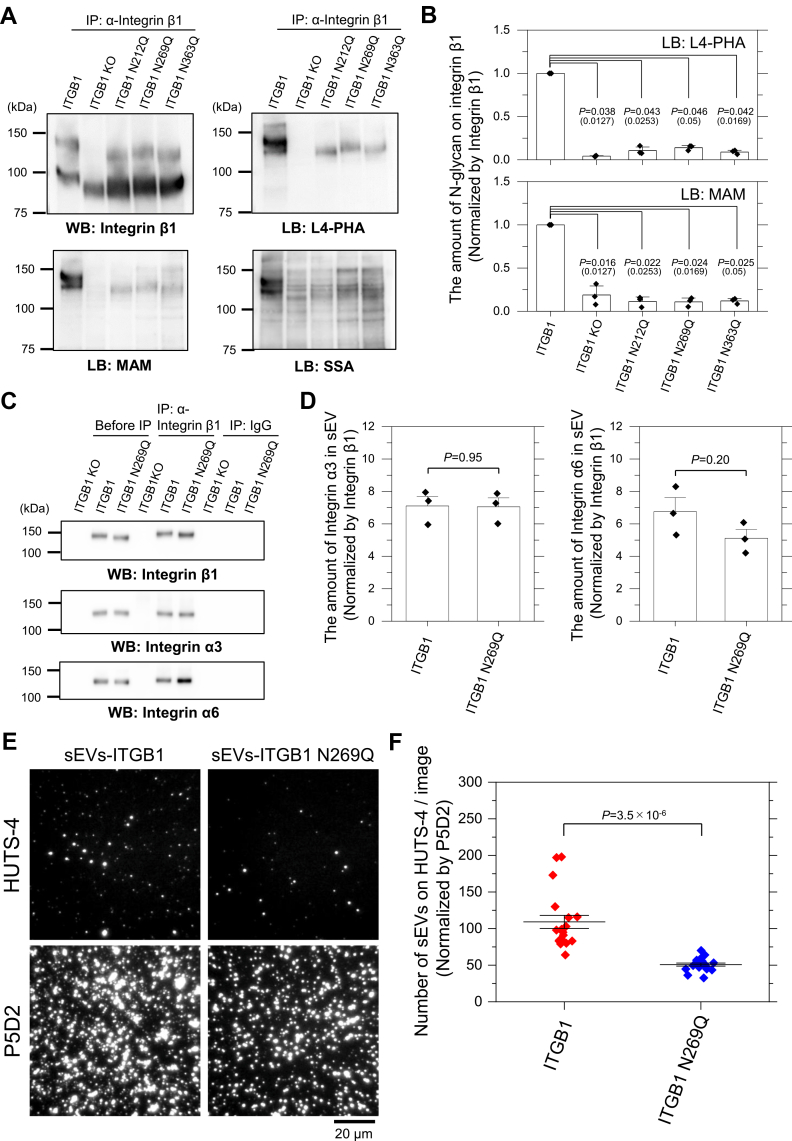
**Glycan at N269 of integrin β1 sustains the active conformation of integrin dimers.***A*, lectin blotting of immunoprecipitated integrin β1 WT and NQ mutants in B16 cells by L4-PHA, MAM, and SSA, which recognize β1-6-branched N-glycan, α2,3-linked sialic acid, and α2,6-sialic acid, respectively. *B*, The lectin blotting of immunoprecipitated integrin β1 WT and NQ mutants in B16 cells was quantitatively analyzed. The results were expressed as ratios relative to the integrin β1 WT level. *C*, immunoprecipitation to examine integrin dimer formation in sEVs derived from integrin β1 KO-, integrin β1-rescued, and the N269Q mutant-expressed B16 cells. Western blot analysis of integrin α3 and α6 in the samples isolated by immunoprecipitation of integrin β1. *D*, quantitative analysis of the amounts of integrin α3 and α6 by western blotting of samples isolated by immunoprecipitation of integrin β1. *E*, fluorescent images of single particles of sEVs derived from integrin β1 WT-rescued and the N269Q mutant-expressed B16 cells, which were attached to anti-integrin β1 HUTS-4 and P5D2 antibodies on glass. Anti-integrin β1 HUTS-4 antibody recognizes only the activated conformation of integrin β1, while anti-integrin β1 P5D2 recognizes both the activated and inactivated one. *F*, The number of sEVs attached to anti-integrin β1 HUTS-4 antibody, which were normalized by those bound to P5D2 (*n* = 19 images). The data are presented as the mean ± SE. *p*-value was calculated by Welch’s *t* test (two-sided). In *B*, because multiple statistical comparisons were required, the significance levels were corrected using the Holm-Sidak method and are indicated in parentheses. sEV, small extracellular vesicle.

As our results indicated that the glycan at N269 of integrin β1 in sEVs enhances their binding affinity to laminin or the number of activated integrin β1 molecules, we aimed to gain further insight into the underlying mechanism. The levels of highly branched complex-type N-glycans, α2,3-linked sialic acid, and α2,6-linked sialic acid on integrin β1 were analyzed by lectin blotting with L4-PHA, MAM, and SSA, respectively, following immunoprecipitation of integrin β1 antibody from the lysates of cells expressing the N212Q, N269Q, or N363Q mutants. Reactivity with these three lectins tended to be weaker for all three mutants compared to integrin β1 WT (Fig. 7, *A* and *B*). This finding indicates that highly branched complex-type N-glycans containing α2,3/α2,6-linked sialic acids were present among the glycans attached to N212, N269, and N363. Subsequently, we examined whether the integrin β1 N269Q mutant alters heterodimerization because integrins function as αβ heterodimers. Consequently, we performed coimmunoprecipitation to determine the amount of integrin α6 and α3 that form heterodimers with integrin β1 N269Q in B16-sEVs. The results demonstrated that equivalent levels of integrin α6 and α3 were detected in both sEVs containing integrin β1 WT and those containing the N269Q mutant after immunoprecipitation with anti-integrin β1 antibody (Fig. 7, *C* and *D*). Thus, these findings demonstrate that the glycan attached to N269 of integrin β1 does not alter the heterodimerization of integrin α3β1 and α6β1 in sEVs.

Finally, we investigated whether the glycan attached to N269 of integrin β1 alters its conformation and subsequently activates integrin β1 because the conformation of integrin heterodimer is closely correlated with the activity (33). We quantified the number of sEVs containing either integrin β1 WT or N269Q mutant bound to the HUTS-4 antibody coated on glass, which specifically recognizes only the activated conformation of integrin β1 (34) (Fig. 7*E*). The number of sEVs bound to the HUTS-4 antibody was normalized by those bound to the P5D2 antibody, which recognizes both the active and inactive forms of integrin β1. The results demonstrated that the number of sEVs containing the integrin β1 N269Q mutant bound to HUTS-4 was 47 ± 4.1% of those containing integrin β1 WT, representing a significantly lower binding affinity or smaller number of activated integrin β1 (Fig. 7*F*, *p* = 3.5 × 10^−6^). These findings suggest that the glycan attached to N269 of integrin β1 induces conformational changes that activate the integrin heterodimer, thereby enhancing the binding ability of B16 cell-derived sEVs to laminin.

## Discussion

Our glycomic analysis revealed that the N-glycans of integrin β1 in tumor cell-derived sEVs were rich in sialic acid and highly branched complex-type N-glycans, whereas oligomannose glycans and less branched and less sialylated N-glycans were predominant in the cells secreting these sEVs (Fig. 1). Simultaneous single-molecule imaging and super-resolution microscopy demonstrated that sialic acid, particularly the α2,6-linked sialic acid produced by ST6GAL1, has a crucial role in enhancing the binding ability of sEVs to laminin (Fig. 3). In addition, N-glycans, especially at N269 of integrin β1 in sEVs, were shown to enhance both the binding affinity of integrin β1 to laminin and the abundance of activated integrin β1 on both glass and recipient cells (Figure 4, Figure 5, Figure 6). Furthermore, it was revealed that the glycan at N269 of integrin β1 did not affect the recruitment of integrin β1 to sEVs and its dimerization with the integrin α subunit; however, it contributes to the activation of the integrin heterodimer (Fig. 7). These results explicitly show that the glycan at N269 of integrin β1 reinforced the integrin- and laminin-mediated binding of tumor cell-derived sEVs to recipient cells.

Highly branched complex-type N-glycans and sialic acids are significantly more abundant in tumor cells than in normal cells (35, 36). Our results demonstrated that the highly branched complex-type N-glycans and α2,6-linked sialic acids of integrin β1 in tumor cell-derived sEVs promoted the binding of sEVs to laminin on both glass and recipient cells. Furthermore, we discovered that these glycan structures were more enriched in sEVs than in tumor cells that secreted them (Fig. 1). Therefore, the N-glycans of integrin β1 were highly concentrated in tumor cell-derived sEVs and may be critical in the recognition of laminin on recipient cells.

Both MGAT5, which catalyzes the formation of highly branched complex-type N-glycans, and ST6GAL1, which adds sialic acid to galactose *via* α2,6-linkage, are known to be highly associated with cancer growth and metastasis (37, 38). It has been demonstrated that highly branched complex-type N-glycans and sialic acids facilitate integrin-mediated elongation (39) and motility (40, 41) of cancer cells on laminin, suggesting that these glycans in cancer cells may activate laminin receptors. Moreover, previous studies by our group and others have revealed that cancer cell-derived sEVs contain high concentrations of MGAT5 and ST6GAL1 (17, 42). These glycosyltransferases most probably continue to function after sEV uptake, leading to increased expression of highly branched complex-type N-glycans and sialic acids. Taken together, these results suggest that highly branched complex-type N-glycans and α2,6-linked sialic acids not only enhance the binding ability of sEVs to laminin on recipient cells but also indicate that sEVs enriched in these glycans may be pivotal in altering the phenotype of the recipient cells (17, 22, 42).

**Figure 8:**
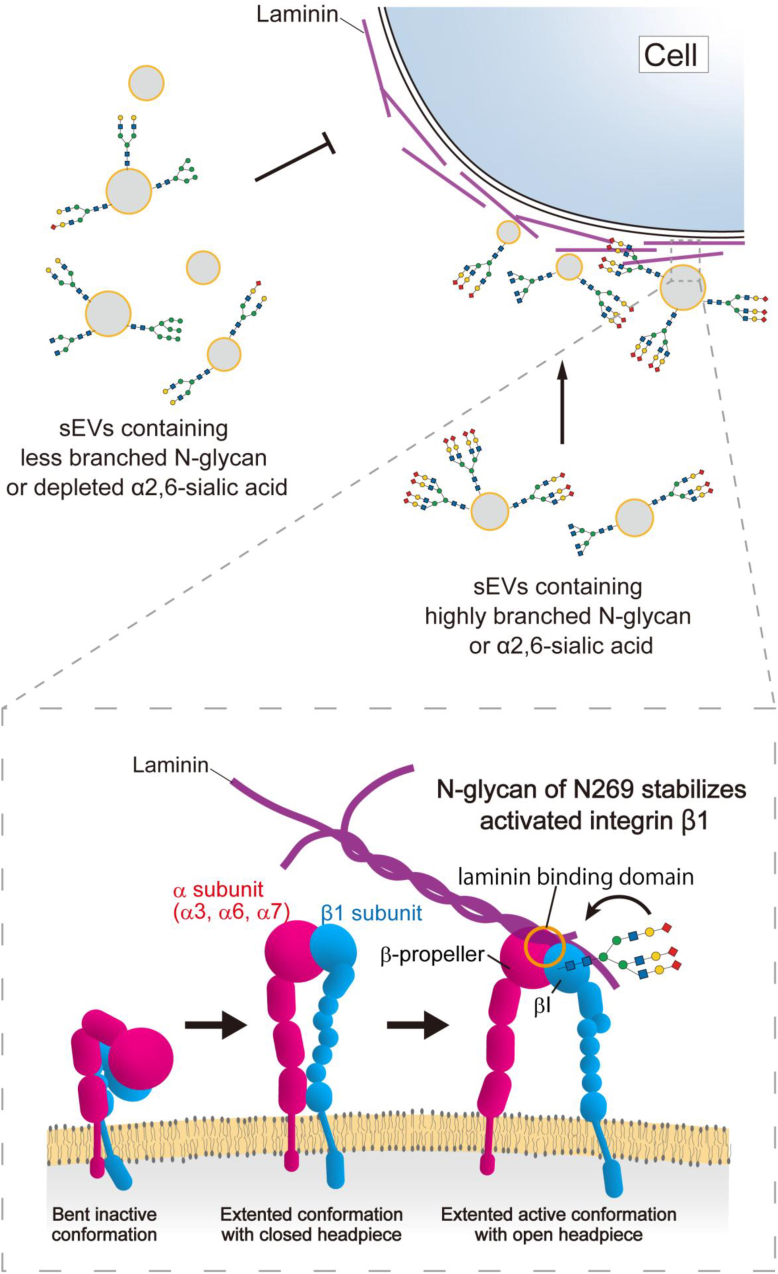
**Schematic diagram of the regulation mechanism of integrin β1 in sEVs to laminin on the recipient cell by N-glycan.** The glycan at N269 is located in the I-like domain of integrin β1.

The regulation of integrin ligand-binding function by N-glycans has been reported as N-glycans, which directly modify integrins, are involved in integrin heterodimer formation in cell PMs (13) and in maintenance of the activated state (14). In addition, previous reports have shown that sialic acid prevents CD82, a suppressor of integrin function, from forming a complex with integrin α3 and α5 (43). The complex formation with CD151, which enhances integrin binding to laminin, is regulated by highly branched complex-type N-glycans on the integrin laminin receptor (39). Therefore, alterations in the structure of N-glycans may modify the complex formations between integrins and the auxiliary molecules, thereby amplifying or attenuating integrin function. In this study, we focused on the N-glycans on integrin β1 and found that the glycan at N269 of integrin β1 enhanced its binding activity in sEVs that interact with laminin (Figure 4, Figure 5, Figure 6). In contrast, the removal of this N-glycan from integrin β1 neither affected the recruitment of integrin β1 to sEVs nor the formation of integrin heterodimers in sEVs, but reduced the active form of integrin β1 (Fig. 7). Consequently, the results of this study revealed that the glycan at N269 of integrin β1 promotes a more active conformation of the integrin heterodimer. In addition, as this glycan is attached to Asn in the I-like domain (rather than near the transmembrane domain), where it interacts with auxiliary molecules (32), it likely directly contributes to the stabilization of the activated conformation of the integrin dimer (Fig. 8). This is supported by the results of the present study, which showed that the anti-integrin β1 HUTS-4 antibody, which recognizes the active conformation (355–425 amino acids in the I-like domain and the hybrid domain of the extended active conformation with an open headpiece) of integrin β1 in heterodimers (Fig. 8), was less frequently bound to the N269Q mutant of integrin β1 than to the integrin β1 WT in sEVs (Fig. 7*F*). Alternatively, the glycan at N269 in the I-like domain of integrin β1 may stabilize the extended active conformation with an open headpiece even before binding to the laminin (Fig. 8).

Previous studies have suggested that N-glycans and sialic acids on sEV are involved in their uptake by recipient cells (22, 44). It has been proposed that Siglecs on recipient cells recognize sialic acids on sEVs, thereby facilitating sEV uptake (18, 45). On the other hand, it has also been reported that the removal of sialic acids from sEVs by sialidase increases their uptake by recipient cells (21, 44). Furthermore, it is suggested that there are subtypes of sEVs that comprise different molecules (46, 47), and each subtype is internalized by varying endocytosis mechanisms (30). Therefore, glycans may differentially regulate the binding and uptake of sEVs by recipient cells depending on the underlying mechanism. Thus, by focusing on integrin- and laminin-mediated binding, we elucidated the molecular mechanisms underlying the regulation of sEV binding to recipient cells by N-glycans. However, these findings were obtained by examining the functions of sEVs derived from B16 cells. It will be important to investigate whether sEVs derived from tumor cells of other tissue origins also bind to recipient cells through the same mechanisms. Furthermore, several previous studies could not explain our results (22, 44). Glycan-lectin and electrostatic interactions by glycans may also be involved in the binding of sEVs to recipient cells, and glycans may regulate sEV uptake by a complex mechanism that combines several mechanisms.

In summary, this study demonstrated that integrin β1 in tumor cell-derived sEVs contains a higher abundance of highly branched complex-type N-type glycans and sialic acids than in tumor cell-secreting sEVs. Direct observation of the sEV binding to laminin on recipient cells using single-particle imaging and super-resolution microscopy explicitly provided insights into these glycans, facilitating integrin-mediated binding of sEVs. As the uptake of sEVs derived from tumor cells is implicated in tumor metastasis, the regulatory mechanisms of sEV binding to laminin by N-glycans, as elucidated in this study, may contribute to more effective cancer therapies.

## Experimental procedures

### Materials

Mouse melanoma cell line (B16) and human lung carcinoma cell line (A549) were purchased from ATCC, and mouse neuroblastoma cell line (Neuro2A) was purchased from RIKEN Cell Bank. Mouse embryonic fibroblast cell line (iMEF) was given by Dr Toshiro Okazaki (48, 49).

All antibodies were obtained from commercial sources. The following primary antibodies were used for western blotting: mouse monoclonal anti-CD29 clone 18 (1:2000, BD Bioscience, Cat# 610467), rabbit polyclonal anti-integrin β1 (1:500, Abcam, Cat# ab183666), goat polyclonal anti-integrin β1 (1:500, R&D, Cat# AF2405), rabbit polyclonal anti-integrin α3 (1:500, Abcam, Cat# ab190731), rabbit polyclonal anti-integrin α6 (1:500, Cell Signaling, Cat# 3750S), mouse monoclonal anti-CD81 B-11 (1:500, Santa Cruz biotechnology, Cat# SC166029), mouse monoclonal anti-β actin 15G5A11/E2 (1:5000, Thermo Fisher Scientific, MA1-140), mouse monoclonal anti-MGAT5 (1:300, R&D, Cat# 706824), mouse monoclonal anti-GAPDH (1:2000, Millipore, Cat# MAB374), mouse monoclonal anti-VDAC1 (1:2000, abcam, Cat# ab14734). Additionally, goat anti-mouse IgG-horseradish peroxidase (HRP) (1:5000, Millipore, Cat# 12-349), donkey anti-rabbit IgG-HRP (1:4000, Cytiva, Cat# NA934), goat anti-rabbit IgG-HRP (1:10,000, Sigma‒Aldrich, Cat# A0545), and donkey anti-goat IgG-HRP (1:10,000, Jackson Cat# 705-035-147) were used as secondary antibodies. For immunoprecipitation, rabbit monoclonal IgG isotype control EPR25A (Abcam, Cat# ab172730), rabbit polyclonal anti-integrin β1 (Abcam, Cat# ab183666), mouse monoclonal IgG1-κ negative control MOPC-21 (Millipore, Cat# MABF1081Z), mouse monoclonal anti-integrin β1 P5D2 (Abcam, Cat# ab24693) were used. In addition, for the quantitative observation of sEVs binding to antibodies and laminin, mouse monoclonal anti-CD81 JS-81 (BD Pharmingen, Cat# 555675), laminin-511 (Biolaminin 511 LN, BioLamina), mouse monoclonal anti-integrin β1 P5D2 (abcam, Cat# ab24693), mouse monoclonal anti-integrin β1 HUTS-4 (Millipore, MAB2079Z) were used.

### Cell culture and transfection

*Mycoplasma* contamination was not detected in any of the cell lines used in this study. B16, A549, Neuro2A, and iMEF cell lines were cultured in Dullbecco’s modified Eagle’s medium (DMEM, Sigma-Aldrich) supplemented with 10% FBS, 100 U/ml penicillin, and 100 μg/ml streptomycin.

WT or integrin β1-KO B16 melanoma cells were cotransfected with 1000 ng of the plasmid for CD81-Halo7tag or integrin β1 WT or NQ mutant together with pCMV-mPBase (neo-) by Lipofectamine 3000 according to the manufacturer’s recommendations. The cell lines that stably express CD81-Halo7tag were selected, using 300 μg/ml (final concentration) of zeocin (50, 51).

### Plasmid construction

Oligonucleotides used for plasmid construction were listed in Table S2. Human CD81 (NM_004356 and pFN21AE5228) cDNA was purchased from Kazusa DNA Res. Inst. The sequence of CD81-Halo7tag (Promega) was constructed in previous study (8) and inserted into the PiggyBac transposon vector (pPB-Zeo) (50, 51). cDNA for WT human integrin β1 (NM_002211) was amplified by PCR and inserted into EcoR1/NotI sites of pPB-Zeo vector by Gibson assembly. Plasmids for integrin β1 mutants lacking one of the N-glycans (N212Q, N269Q, or N363Q) were generated by PCR using pPB-Zeo/integrin β1 as a template and QuikChange Lightning Site-Directed Mutagenesis Kit (Qiagen). To construct the plasmids for gene editing, pX330-puro was digested with BbsI, followed by ligation with the annealed oligonucleotides listed in Table S2.

### KO of glycosyltransferase and integrin β1 in B16 cells

To generate *Mgat5* KO, *St6gal1* KO and *Itgb1* KO cell clones, two different pX330-puro plasmids encoding sgRNAs targeting each gene were transfected using lipofectamine 3000. One day after transfection, cells were selected with 3 μg/ml puromycin (for *Mgat5* KO and *St6gal1* KO) or 5 μg/ml puromycin (for *Itgb1* KO), and the surviving cell clones were isolated by limiting dilution. Primers for genotyping PCR were listed in Table S2.

### MGAT5 activity assay

MGAT5 activity was measured as described previously (52). In brief, cell lysates were incubated in 10 μl of reaction buffer [125 mM MES-NaOH (pH 6.2), 10 mM EDTA, 200 mM GlcNAc, 0.5% Triton X-100, and 1 mg/ml BSA] containing 20 mM UDP-GlcNAc and 10 μM acceptor substrate (GnGnbi-PA) at 37 °C. As a positive control, recombinant soluble human MGAT5 (from Thr121 to Leu741) purified from COS7 cells was also used (53). After enzymatic reactions, samples were heated at 99 °C for 2 min, and 40 μl of water was added. After centrifugation at 21,500×*g* for 5 min, the supernatants were analyzed by reverse-phase HPLC with an ODS column (4.6 × 250 mm; Inertsil ODS-3, GL Sciences).

### Flow cytometry

Cells were washed with PBS twice and collected using cell scrapers, followed by centrifugation at 1400×*g* for 3 min. The cells were washed with FACS buffer (1% BSA, 0.1% NaN_3_, in PBS) once and stained with SNA-FITC (1:200, Vector Laboratories, Cat# FL-1301) in FACS buffer on ice for 15 min. Data were collected with a FACS Melody cell sorter and analyzed using FlowJo software (BD Biosciences; https://www.flowjo.com).

### Isolation of sEVs

For glycomics, sEVs were isolated as described previously (17). Briefly, culture media of B16 cells were centrifuged at 1200*g* for 5 min, concentrated with Amicon-Ultra filter units (10 kDa, Millipore), and centrifuged at 10,000*g* for 30 min. The supernatants were further ultracentrifuged at 100,000*g* for 60 min, and the resultant pellets were used as sEVs.

sEVs were purified as previously described for single-particle observation (8). Cells that secreted sEVs were cultured either in two 100-mm dishes for the observation of sEVs on glass or in two 150-mm dishes for western blotting analysis and the observation of sEVs on cell PMs. The cells were grown to approximately 80% confluency (1 × 10^8^ cells/150-mm dish). The cell culture medium in the 100-mm or 150-mm dishes was replaced with 10 ml or 30 ml of FBS-free medium, respectively. After 48 h of incubation, the cell culture supernatant was then centrifuged at 300×*g* for 10 min at 4 °C to remove cells. Subsequently, the supernatant was further centrifuged at 2000×*g* and 4 °C for 10 min to remove sediment and eliminate apoptotic bodies. Then, the supernatant was centrifuged at 10,000×*g* and 4 °C for 30 min (himac CF16RN, T9A31 angle rotor, 50 ml Falcon tube) to remove microvesicles. Finally, the supernatant was concentrated by ultrafiltration using an Amicon Ultra 15 100K (Millipore) or a Centricon Plus 70 100K (Millipore) filter unit. The collected sEVs were incubated with 100 nM (final concentration) HaloTag TMR ligand (Promega) or HaloTag SF650T ligand (Goryo Chemical) for 1 h at 37 °C. Moreover, the sEVs were treated with 50,000 U/ml PNGaseF (New England Biolabs), 2000 U/ml α2-3,6,8 sialidase (P0720, New England Biolabs), or 800 U/ml α2,3-sialidase (New England Biolabs) for 1 h at 37 °C. The mock experiments were performed by incubating sEVs under the same conditions without these enzymes. The sEVs were then pelleted by ultracentrifugation at 200,000×*g* and 4 °C for 4 h (himac CS100FNX with an S55A2 angle rotor and S308892A microtubes for ultracentrifugation). The resulting pellet was resuspended in Hank’s balanced salt solution (HBSS) for microscopic observation. For western blotting, the pellet was suspended in RIPA buffer containing protease inhibitor cocktail set III (Millipore).

### Transmission electron microscopy of sEVs after negative staining

A copper grid with a carbon-coated acetylcellulose film (EM Japan) was washed briefly (∼1 min). 10 microliters of water was placed on the grid and then blotted to eliminate the excess liquid. Next, 5 μl of the sEV sample was pipetted onto the grid and incubated for 1 min. The excess liquid was then removed by blotting. The grid was then briefly (∼45 s) treated with 5 μl of 2% phosphor tungstic acid (TAAB Laboratory and Microscopy), followed by blotting to remove excess liquid. Next, the grid was air-dried at room temperature for 3 days in a desiccator containing silica gel desiccant and subsequently subjected to transmission electron microscope observation. sEV images were obtained using a single transmission electron microscope instrument (JEM-2100F, JEOL) at 200 kV. These images were produced by computing the mean of 3-s acquisitions captured by a side-mounted CCD camera (Gatan) and processed using Digital Micrograph software (Gatan; https://www.gatan.com/products/tem-analysis/digitalmicrograph-software).

### Glycomics using LC-ESI MS

sEVs or cells were lysed with TBS containing 1% Nonidet P-40 and protease inhibitors. After centrifugation at 15,000×*g* for 10 min, integrin β1 was immunoprecipitated with rabbit anti-integrin β1 pAb (Abcam, Cat# ab183666) and Dynabeads protein G with overnight rotation. After washing the beads with TBS containing 0.1% Nonidet P-40 three times, the bound proteins were eluted with SDS sample buffer. After SDS-PAGE and protein transfer to a PVDF membrane, the membrane was washed once with ethanol for 1 min, followed by wash with water for 1 min three times prior to staining for 5 min with Direct Blue 71 (0.8 ml of solution A: 0.1% (w/v) Direct Blue 71 [Sigma–Aldrich] in 10 ml of solution B: acetic acid:ethanol:water = 1:4:5). After destaining the membrane with solution B for 1 min, the membrane was dried at room temperature for 16 h. The bands corresponding to integrin β1 were excised, placed into a 96-well plate, incubated in 100 μl of 1% (w/v) poly(vinylpyrrolidone) 40,000 in 50% (v/v) methanol for 20 min with agitation, and washed with water (100 μl × 5 times).

*N*-glycans were released by PNGase F (Roche), converted to alditol *N*-glycans by reduction, and desalted as described previously (54). Alditol *N*-glycans were separated on a carbon column (5 mm HyperCarb, 1 mm I.D. x 100 mm, Thermo Fisher Scientific) using a Vanquish HPLC pump (flow rate, 50 μl/min; column oven, 40 °C, solvent A (10 mM ammonium bicarbonate), solvent B (90% (v/v) acetonitrile in 10 mM ammonium bicarbonate) under the following gradient conditions involving a sequence of isocratic and two segmented linear gradients: 0 to 8 min, 100% of solvent A; 8 to 38 min, 7.5 to 17.4% of solvent B; 38 to 73 min, 17.5 to 45% of solvent B, increasing to 90% of solvent B for 7 min. They were then reequilibrated with 100% of solvent A for 15 min. The eluate was continuously introduced into an ESI source (Thermo Fisher Scientific).

About the mass spectrometer (LTQ Orbitrap XL, Hybrid Linear Ion Trap-Orbitrap mass spectrometer, Thermo Fisher Scientific), the voltage of the capillary source was set at 3 kV, and the temperature of the transfer capillary was maintained at 300 °C. The capillary voltage and tube lens voltage were set at −18.0 V and −112.8 V, respectively. MS spectra were obtained in the negative polarity using an Orbitrap (mass range, m/z 500–2500; resolution, 15,000; mass accuracy, 3 ppm), and MS/MS spectra were obtained using an Ion Trap (data-dependent top 3, collision-induced dissociation). Monoisotopic masses of glycans observed *via* MS were analyzed to find possible monosaccharide compositions using the GlycoMod tool available on the ExPASy server (http://au.expasy.org/tools/glycomod; mass tolerance for precursor ions, ±0.01 Da), and the proposed glycan structures were selected from the GlyConnect database (https://glyconnect.expasy.org/) linked *via* GlycoMod and according to the separation pattern on a carbon column as reported previously (55). The linkages of sialic acid in biantennary glycans were assigned according to the standard glycans derived from bovine fetuin (54). The peak intensities of alditol *N*-glycans were calculated on the extracted ion chromatogram using Xcalibur software version 2.2 (Thermo Fisher Scientific; https://tools.thermofisher.com/content/sfs/manuals/Man-XCALI-97211-Xcalibur-22-Qual-ManXCALI97211-D-EN.pdf). The relative abundances (%) of alditol *N*-glycan structures were calculated by setting the total peak intensities of all detected alditol *N*-glycans in each extracted ion chromatogram to 100%.

### Western and lectin blotting

The cells and sEVs were suspended in RIPA buffer containing protease inhibitor cocktail set III (Millipore). The protein concentrations of the samples were determined using a Thermo BCA protein assay kit (Thermo Fisher Scientific) and adjusted to 1 mg/ml for SDS‒PAGE. Then, 5 × concentrated Laemmli’s SDS sample buffer was added to the samples, and the mixture was incubated at 95 °C for 5 min in a blocking incubator. Next, 10 μl of this mixture was loaded into the lanes of a precast 4 to 12% gradient polyacrylamide gel (4–12% Bolt Bis-Tris Plus Gels, Thermo Fisher Scientific). Molecular weights were determined using Precision Plus Protein Prestained Standards (Bio-Rad Laboratories). After electrophoresis, the proteins were transferred onto a 0.45-μm polyvinylidene difluoride membrane (Millipore). Next, after blocking for 30 min at room temperature and washing, the membrane was incubated with the primary antibody in Tris-buffered saline (20 mM Tris and 150 mM NaCl, pH 7.4) (TBS) supplemented with 0.1% Tween 20 (TBS-T) containing 5% nonfat milk or Blocking One (Nacalai Tesque) overnight at 4 °C. After washing with TBS-T, the membranes were incubated with HRP-conjugated secondary antibody in TBS-T containing 5% nonfat milk or Blocking One solution at room temperature for 1 h. After washing with TBS-T, the membranes were treated with either the ECL start reagent (GE Healthcare) or the ECL select reagent (GE Healthcare) according to the manufacturer’s guidelines. The chemiluminescent images of the membranes were acquired using FUSION-SOLO.7S (Vilber-Loumat) and analyzed using ImageJ.

For lectin blotting, nitrocellulose membranes were used. After protein transfer, the membranes were blocked with TBS-T containing 1% bovine serum albumin (BSA) at 4 °C overnight. The membranes were incubated with HRP-conjugated L4-PHA (J112, MGC Woodchem), MAM (J110, MGC Woodchem), or SSA (J118, MGC Woodchem) in TBS-T containing 1% BSA. Labeling of lectin with HRP was performed using Peroxidase Labeling Kit-NH2 (Dojindo). For SNA blotting, the membranes were blocked with TBS-T, incubated with biotinylated SNA (B-1305, Vector Laboratories) in TBS-T, and subsequently incubated with ABC Standard Kit (PK-4000, Vector Laboratories) in TBS-T.

### Immunoprecipitation

The initial step involved treating 0.6 mg of Protein G-conjugated Dynabeads (Invitrogen) with 200 μl of 15 μg/ml anti-integrin β1 antibody (Abcam, Cat# ab183666) or rabbit IgG isotype control in PBS containing 0.02% Tween 20. The mixture was incubated with rotation for 10 min at room temperature, and then the supernatant was removed. The beads were washed once with PBS containing 0.02% Tween 20 and twice with 20 mM sodium phosphate (pH 7.0) and 150 mM NaCl using a magnetic rack. Then, the antibody-conjugated beads were resuspended in 5 mM BS3 (bis(sulfosuccinimidyl)suberate, Thermo Fisher Scientific, Cat#21580), 20 mM sodium phosphate (pH 7.0), and 150 mM NaCl for crosslinking between the antibody and the beads. After incubation with rotation for 30 min at room temperature, 12.5 μl of 1 M Tris-HCl (pH 7.5) was added to the solution, which was then incubated with rotation for an additional 15 min at room temperature. Subsequently, the supernatant was removed, and the beads were washed once with PBS containing 0.02% Tween 20 and then twice with PBS using a magnetic rack. For Figure 2, *E* and *F*, the crosslinking step was skipped.

The antibody-conjugated beads were mixed with 300 μl of B16-WT/ITGB1KO/ITGB1/ITGB1N269Q-derived sEVs lysate (0.167 mg/ml protein concentration) in PBS or 250 μl of cell lysates in TBS containing 1% Nonidet P-40. After the mixture was incubated with rotation for 1 h, the supernatant was removed, and the beads were washed three times with the lysis buffer. Then, the beads were resuspended in Laemmli’s SDS sample buffer and incubated at 70 °C for 10 min. The supernatant was collected using a magnetic rack and analyzed by western blotting.

### Determination of the concentration of sEVs by TIRF microscopy

The concentrations of protein and lipids in the sEV suspension (on the order of nM) were too low to be determined quantitatively *via* conventional spectrophotometry. Therefore, we employed single-fluorescent particle tracking to directly quantify the number of sEVs. In brief, the glass windows of triple-well glass-base dishes were coated with 50 μl of 10 μg/ml anti-CD81 IgG antibody (JS-81, BD Biosciences) in HBSS and then incubated for 2 h at 37 °C. Subsequently, the antibody solution was removed, and the glass window was coated with either 50 μl of 50 μg/ml casein (Sigma‒Aldrich) in HBSS for 1 h at 37 °C. This coating process was essential for mitigating the nonspecific binding of sEVs to the glass surface during subsequent experiments.

Fifty μl of three distinct concentrations of sEVs containing CD81-Halo7 conjugated with SF650T in HBSS was applied to glass windows pre-coated with antibodies against CD81. The samples were then incubated at 37 °C for 1 h. After removing the sEVs and washing twice with HBSS, the individual fluorescent particles of the sEVs were observed at 37 °C with single-molecule detection sensitivity by total internal reflection fluorescence microscopy (TIRFM) using an Olympus IX-83 microscope (60x 1.49 NA oil objective) equipped with a high-speed gated image intensifier (C9016-02MLG; Hamamatsu Photonics) coupled to an sCMOS camera (ORCA-Flash4.0 V2; Hamamatsu Photonics), as previously described (56, 57, 58). SF650T was excited using a 647-nm laser (LuxXPlus647-140, 140 mW, Omicron Laserrange) at an intensity of 0.3 μW/μm^2^.

All the acquired sEV movies were analyzed using ImageJ (Fiji). First, a noise reduction process was carried out, wherein 10 frames of sEV images observed at 30 frames/sec were averaged. Subsequently, each pixel was replaced by the average of the 3 × 3 neighborhood pixels using a smooth filter. Single particles of sEVs whose fluorescence intensity exceeded that of free SF650T were identified, and the numbers of sEVs were counted. By performing these observations at three distinct sEV concentrations, we obtained a calibration curve. Thereafter, the concentrations of sEVs derived from both intact and integrin subunit-KO cells were adjusted to equal levels. For analyses of sEV binding to laminin-coated glass surfaces, the concentration was set to 0.8 × 10^10^ particles/ml, whereas for analyses of sEV binding to iMEF cells, it was set to 4 × 10^10^ particles/ml.

### Determination of the number of sEVs bound to laminin on glass

To prevent nonspecific binding of sEVs to the glass surface, 50 μl of 50 μg/ml casein solution in HBSS was added to glass windows in single-well or triple-well glass-base dishesand incubated for 1 h at 37 °C. Then, the casein solution was replaced with 50 μl of 20 μg/ml laminin-511 (BioLamina) or 50 μg/ml casein (Sigma‒Aldrich) for 2 h at 37 °C. After removal of the ECM or casein solution, 50 μl of sEVs containing CD81-Halo7 labeled with SF650T at the same concentration was applied to glass and incubated for 1 h at 37 °C. After removal of the sEV suspension and two washes with HBSS, individual fluorescent particles of the sEVs in HBSS were visualized at 37 °C with single-molecule detection sensitivity by TIRFM. The number of sEVs bound to the ECM or casein on glass was determined using ImageJ (Fiji) as described above. The number of sEVs bound to ECM-coated glass was estimated by subtracting the number of sEVs bound to casein-coated glass in a 1024 pixel × 1024 pixel (81.9 μm × 81.9 μm) image.

### Normalization of the HUTS-4 binding signal to the P5D2 binding signal

In this study, we compared the amount of sEVs containing active integrin β1. For this comparison, it is essential that the total amount of sEVs containing integrin β1 be equivalent. P5D2 recognizes both the active and inactive forms of integrin β1. We therefore quantified the amount of sEV-CD81-Halo7-TMR binding to P5D2 on glass as a measure of the total sEV population containing integrin β1. In parallel, the binding of sEVs to HUTS-4, an antibody that specifically recognizes the active form of integrin β1, was measured as the amount of sEVs containing active integrin β1. Normalizing the HUTS-4 binding signal to the P5D2 binding signal allowed us to directly compare the relative abundance of sEVs containing active integrin β1.

### Determination of the number of sEVs bound to cell membranes

sEVs containing CD81-Halo7 labeled with SF650T (sEV-CD81Halo7-SF650T) were incubated with iMEF cells expressing GFP in the cytosol at 37 °C. sEV-CD81Halo7-SF650T was visualized with single-molecule detection sensitivity by TIRFM, and GFP was observed by oblique illumination. The number of sEVs on the cell PM (the cell contour was determined using binarized GFP images) was quantified every 10 min by ImageJ (Fiji) as described above.

### Simultaneous dual-color observation of a dSTORM movie of laminin and single fluorescent particles of sEVs on living cell PMs

For immunostaining of laminin for dSTORM movie observation, 0.67 μl of 5.6 μg/ml SaraFlour650B (SF650B, Goryo Chemical) NHS ester was incubated with 100 μl of 1 μg/ml anti-rabbit IgG antibody (0212-0081, Cappel) in 0.1 M NaHCO_3_ for 60 min at room temperature. SF650B is a spontaneously blinking dye suitable for dSTORM observation in living cells. SF650B-conjugated anti-rabbit IgG was then isolated using a Sephadex G-25 column (GE Healthcare). iMEF cells cultured on glass-based dishes for 2 days were subsequently incubated with 10 μg/ml anti-laminin IgG (L9393; Sigma‒Aldrich) for 30 min at 37 °C. After washing twice with HBSS, the cells were incubated with 10 μg/ml SF650B-conjugated anti-rabbit IgG antibody for 30 min at 37 °C. After washing with HBSS 3 times, sEVs containing CD81-Halo7-TMR were incubated with the cells for another 30 min at 37 °C. Individual fluorescent particles of the sEVs and single molecules of SF650B on the apical surface of the cells at 512 × 512 pixel (25.6 μm × 25.6 μm) were observed at 5-ms resolution (200 frames/second) for 1998 frames by oblique-angle illumination using a Nikon Eclipse Ti inverted microscope (100 × 1.49 NA oil objective) equipped with two sCMOS cameras (ORCA-Fusion; Hamamatsu Photonics). TMR and SF650B were excited using a 561-nm laser (Cobolt Jive 300–561 nm, 300 mW, Cobolt) and a 642-nm laser (LuxX+642-140, 140 mW, Omicron) at 2 and 16 μW/μm^2^, respectively.

dSTORM image reconstructions with a pixel size of 10 nm were performed using the ThunderSTORM plugin for ImageJ (59) installed in the Fiji package (60). Gaussian rendering with a localization precision of 24 nm was utilized in this process.

The dSTORM super-resolution video data were generated using frame information and the x and y coordinates of all the spots, which were incorporated into the csv data output by ThunderSTORM. Furthermore, we used the uncertainty that represents the spatial precision of the spot. A Gaussian distribution was generated for each spot, with the x and y coordinates serving as the center and the SD being equivalent to 6 times the uncertainty of the spot. This distribution indicates the existence probability of the spot. The existence probability distribution for all structures at time t was obtained as a summation of all the distributions for spots that appeared in [6t + 1 6t + 1002] frames. The dSTORM super-resolution video data were generated by repeating this process for time t = 0,1,2,… If the value of each time and coordinate in the dSTORM video data exceeded a threshold value θ, we considered a structure to exist at the time and coordinate. The optimal threshold value θ was determined for each dSTORM video data point to minimize the average in-class variance for the two classes when every value in the dSTORM video data was classified into two classes using the threshold value θ (49, 61, 62). To synchronize the dSTORM movie of laminin structures with the single-particle movie of sEVs, the temporal resolution of the single-particle movie was converted to 33.3 frames/s by averaging 6 frames, and the averaged single-particle image was merged with the dSTORM image created from 1002 frames, of which the middle frame was synchronized with the averaged single-particle image. Ultimately, we created pseudo-real-time movies of laminin and sEVs with a time resolution of 33.3 frames/s over a 5-s duration (167 frames). Single-particle tracking in the movie was performed by in-house computer software based on previously reported methods (63, 64). More details have been described previously (65, 66, 67, 68, 69, 70).

For colocalization analysis, the distances between the centroids of the sEVs and the boundaries of the laminin structures were quantified using single-particle tracking and binarized dSTORM movies. To generate binarized images of the laminin structures, we employed the kernel density estimation method (62). Custom MATLAB software (https://www.mathworks.com/products/matlab.html) was used to perform kernel density estimation binarization of the dSTORM images and measure the distances between single-particle tracking of sEVs and the edges of the binarized image. Detailed information has been described in previous studies (8, 30). When an sEV particle was within an ECM structure, the distance was expressed as a negative value. Furthermore, we simulated the distances between random coordinates and the boundaries of ECM structures *in silico* and calculated their corresponding relative frequency distributions using a bin width of 50 nm. To normalize the relative frequency of sEVs within each bin, we divided the data by the corresponding relative frequency of random spots. A ratio exceeding 1 indicated a higher incidence of sEVs at the measured distance.

## Data availability

Glycomic raw data for glycan structure analysis by LC-ESI MS have been deposited in GlycoPOST (announced ID: GPST000538). All the other data can be found within the article and its supplementary information.

## Supporting information

This article contains supporting information.

## Conflict of interest

Y. K. is an editorial board member of the Journal of Biological Chemistry. Other authors declare no competing or financial interests.
