# Supplementary material for: Highly branched complex-type N-glycans on integrin β1 in extracellular vesicles enhance the binding of sEVs to laminin on recipient cells
Source: J Biol Chem. 2026 May 14;302(6):113146. doi: 10.1016/j.jbc.2026.113146 (PMC13276562; doi:10.1016/j.jbc.2026.113146)
Supplement: Supplemental Tables Figures [file mmc2.docx]

Supporting Information

**Highly branched complex-type N-glycans on integrin β1 in extracellular vesicles enhance the binding of sEVs to laminin on recipient cells**

**Tatsuki Isogai, Yuko Tokoro, Miyako Nakano, Koichiro M. Hirosawa, Rinshi S. Kasai, Yasunari Yokota, Yasuhiko Kizuka*,** **Kenichi G. N. Suzuki***

**^*^**Correspondence: Yasuhiko Kizuka, Ph. D., kizuka.yasuhiko.k8@f.gifu-u.ac.jp

Kenichi G. N. Suzuki, Ph.D., [suzuki.kenichi.b7@f.gifu-u.ac.jp](mailto:suzuki.kenichi.b7@f.gifu-u.ac.jp)

This Supporting information includes:

Figs. S1–S3 (included in this PDF)

Table S1-S2 (S1 is separate file. S2 is included in this PDF)

Movie S1-S5 are separate files.


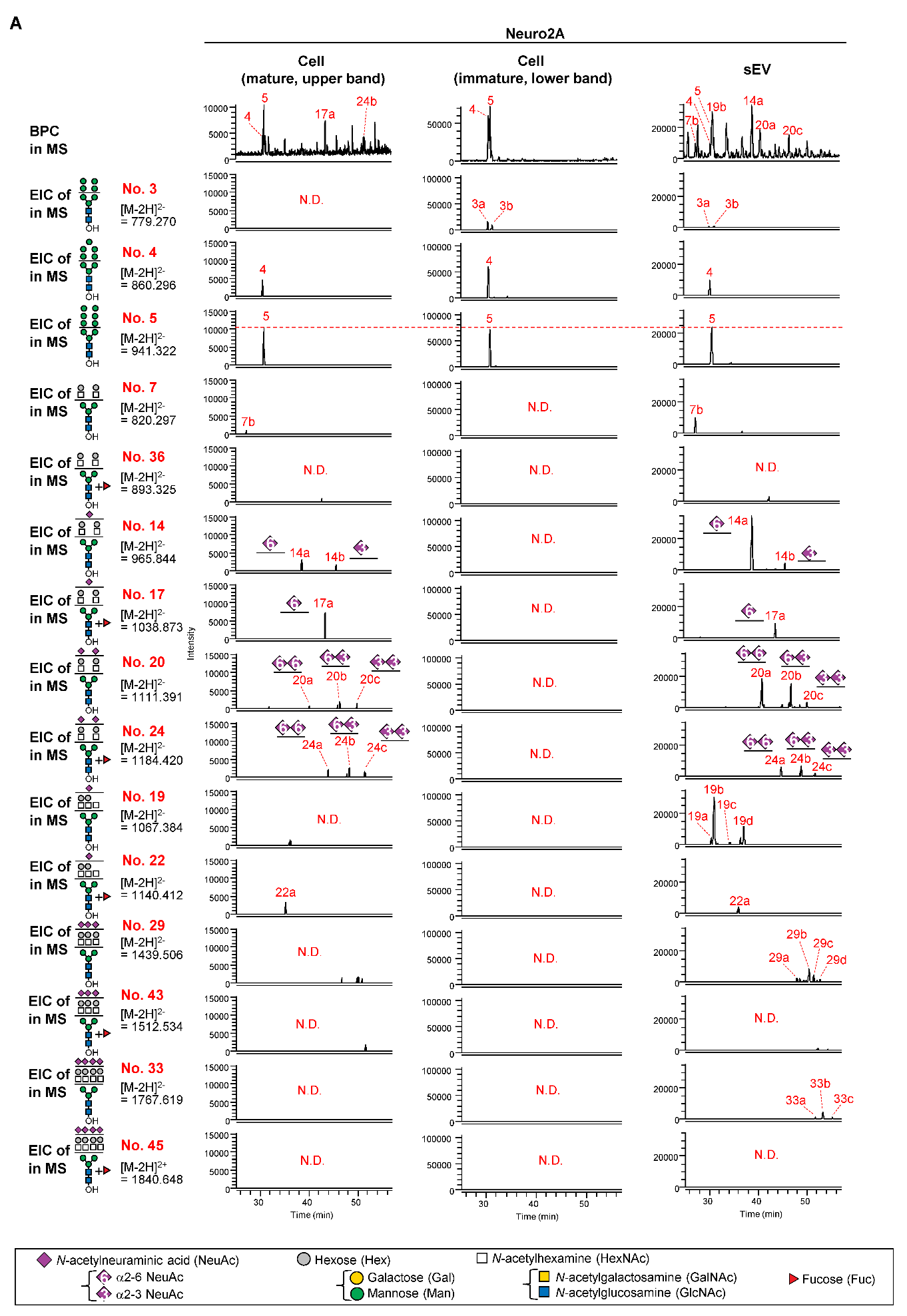
Figure S1


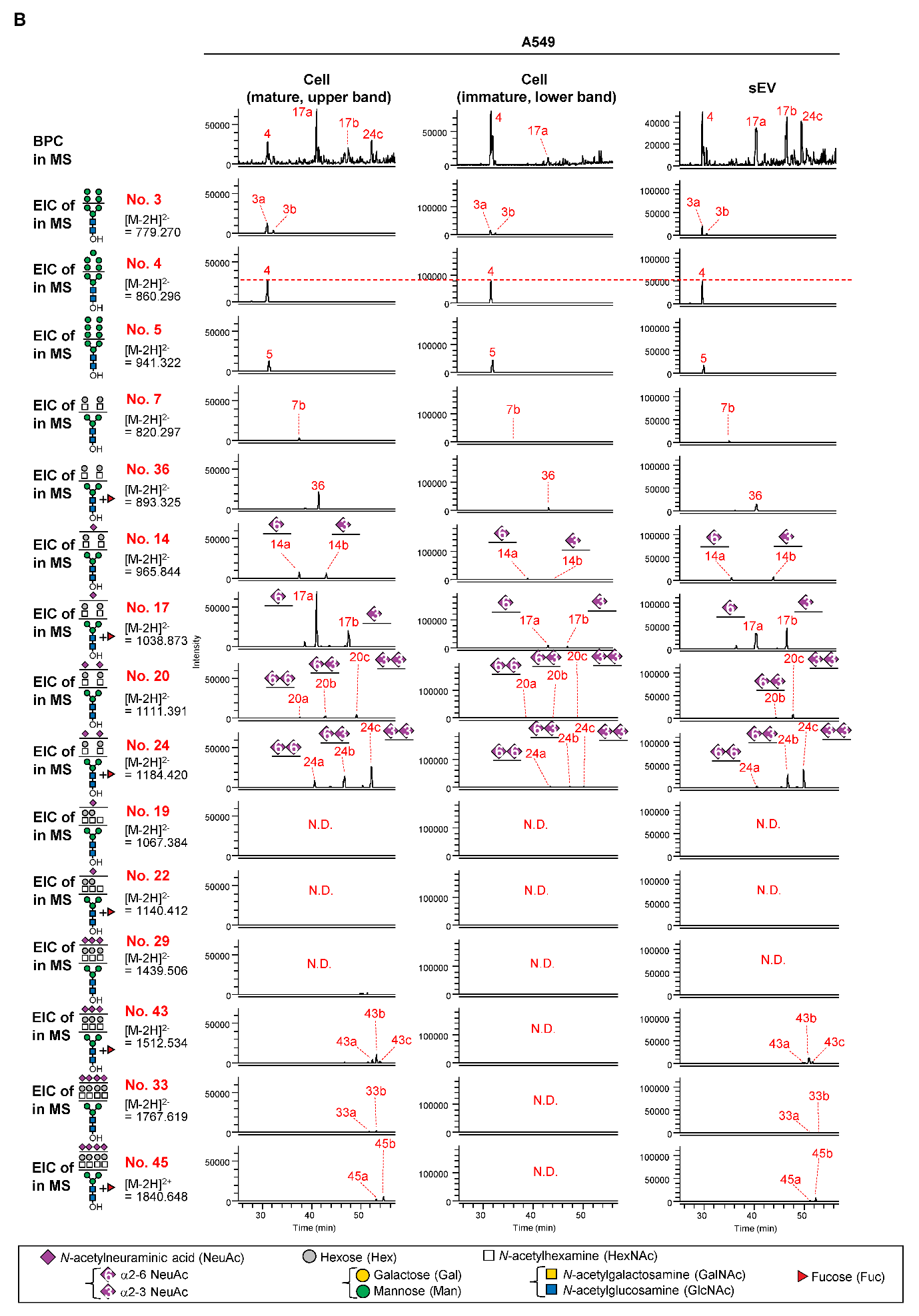


**
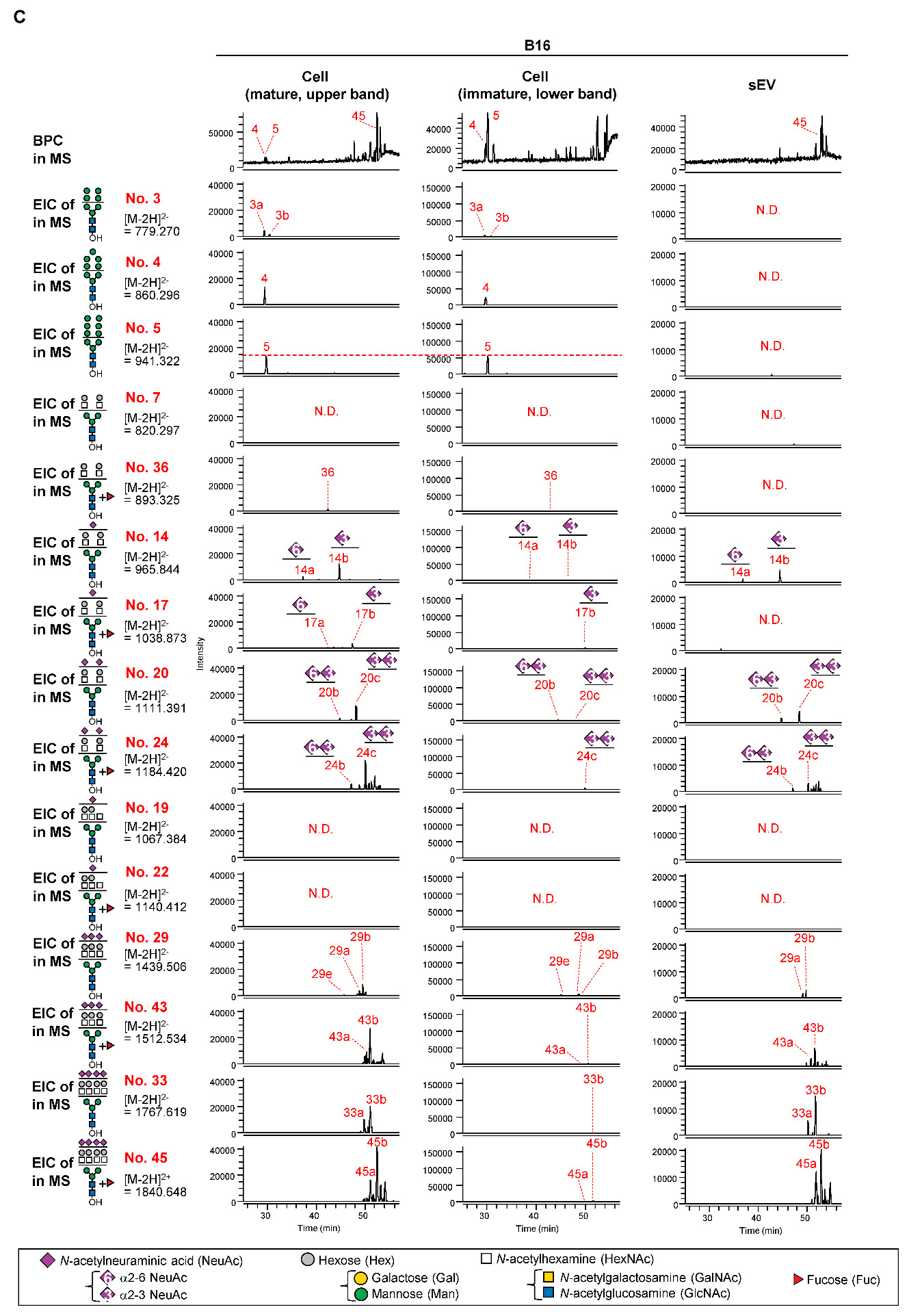
Figure S1. LC-MS N-glycomic analysis of integrin β1 derived from Neuro2A, A549, and B16 sEVs and cells. (*A*-*C*)** Base peak chromatogram (BPC) and extracted ion chromatograms (EICs) of major 15 N-glycans released from immunoprecipitated integrin β1 from cells and sEVs of Neuro2A (A), A549 (B), and B16 (C) cells.

Figure S2


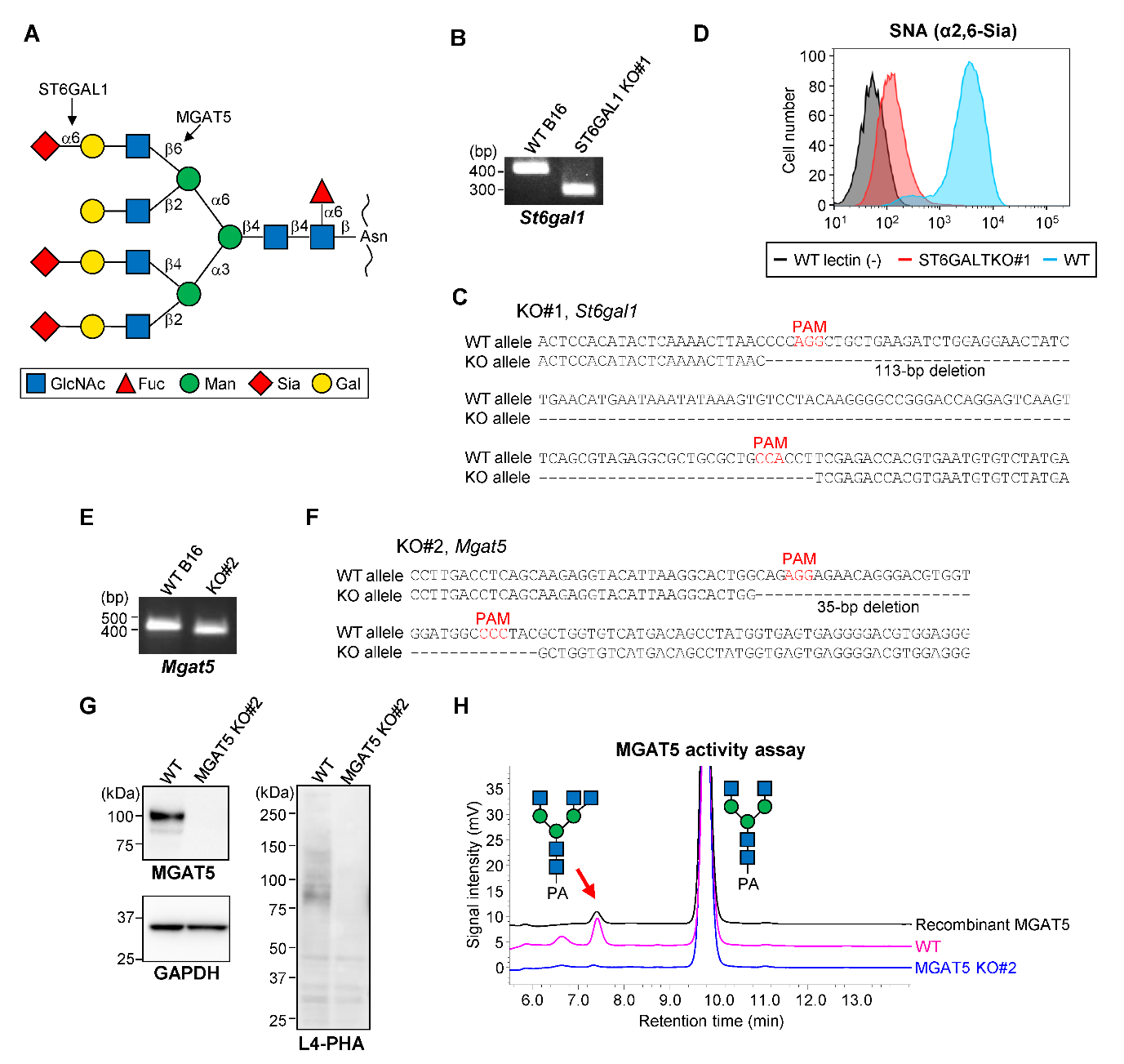


**Figure S2. Establishment of MGAT5 KO and ST6GAL1 KO B16 cells. *A***, Schematic illustration of a sialylated branched N-glycan structure and the actions of ST6GAL1 and MGAT5. ***B***, Genotyping PCR of B16 WT and ST6GAL1 (*St6gal1*) KO B16 cells. ***C***, Genome sequence of the targeted region in ST6GAL1 KO B16 cells. ***D***, B16 WT and ST6GAL1 KO cells were subjected to flowcytometry analysis with SNA-FITC. ***E***, Genotyping PCR of B16 WT and MGAT5 KO B16 cells. ***F***, Genome sequence of the targeted region in MGAT5 KO B16 cells. ***G***, Lysates of B16 WT and MGAT5 KO cells were blotted with anti- MGAT5, anti-GAPDH, and L4-PHA. ***H****,* Cell lysates B16 WT or MGAT5 KO were incubated with a PA-labeled N-glycan to measure the MGAT5 activity. The reaction mixtures were analyzed by reverse-phase HPLC.

Figure S3


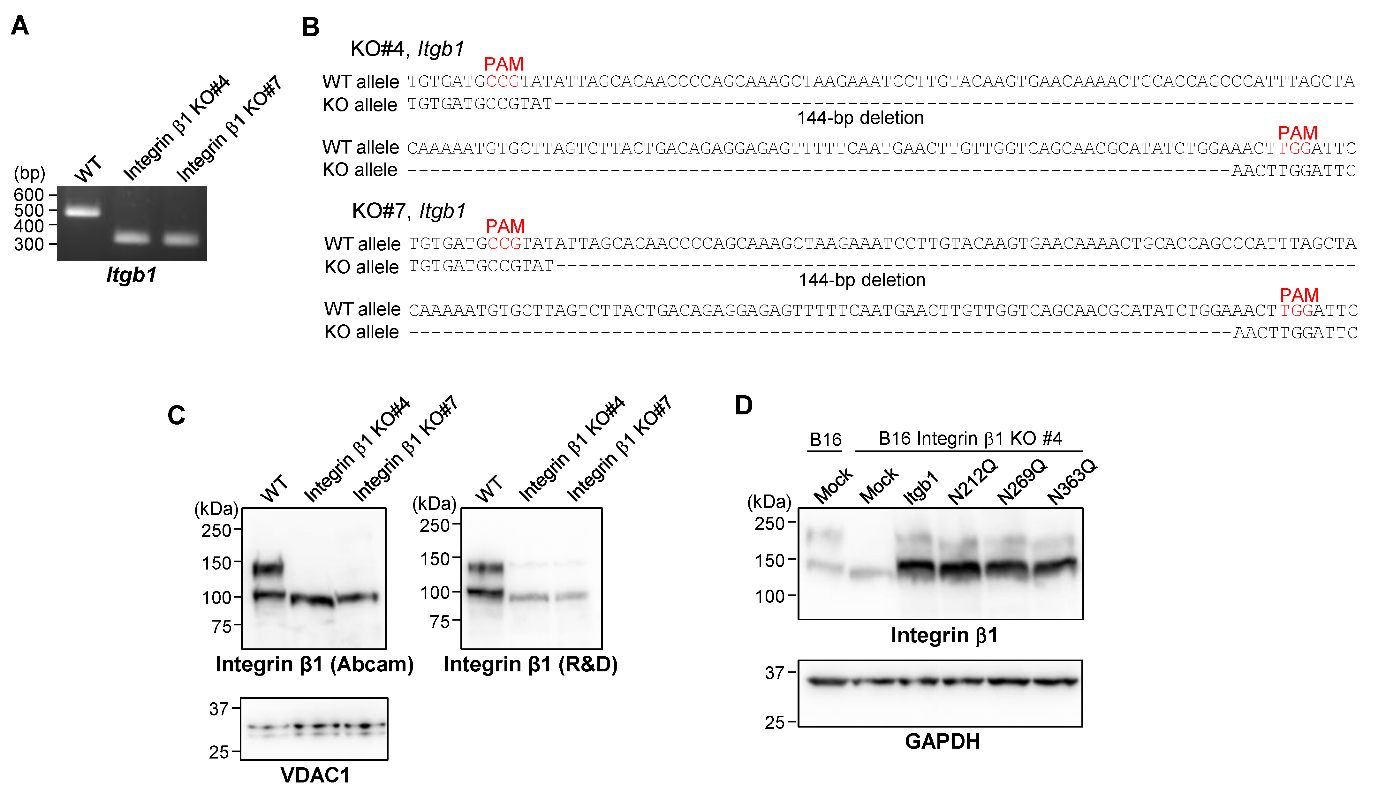


**Figure S3. Generation of B16 integrin b1 KO cells and stable transfectants. *A*,** Genotyping PCR of B16 WT and integrin β1 (*Itgb1*) KO B16 cells. ***B***, Genome sequence of the targeted region in integrin β1 KO B16 cells. ***C***, Lysates of B16 WT and integrin β1 KO cell clones were blotted with anti-integrin β1 and anti-VDAC1. ***D***, Lysates of B16 WT and integrin β1 KO cell clones stably expressing integrin β1 WT or NQ mutant were blotted with anti-integrin β1 and anti-GAPDH.

Table S2

Primers used in this study.

| **Oligonucleotide_Name** | **Sequence** |
| --- | --- |
| Integrin-beta1 WT For | tggcaaagaattgggatccggccaccATGAATTTACAACCAATTTT |
| Integrin-beta1 WT Rev | agttagttgactctagagcggccTCATTTTCCCTCATACTTCGGAT |
| Integrin-beta1 N212Q For | CCCTTGCACAAGTGAACAGCAATGCACCAGCCCATTTAGCT |
| Integrin-beta1 N212Q Rev | AGCTAAATGGGCTGGTGCATTGCTGTTCACTTGTGCAAGGG |
| Integrin-beta1 N269Q For | ATCACTGATTGGCTGGAGGCAAGTTACACGGCTGCTGGTGT |
| Integrin-beta1 N269Q Rev | ACACCAGCAGCCGTGTAACTTGCCTCCAGCCAATCAGTGAT |
| Integrin-beta1 N363Q For | AGTAGGAACATTATCTGCCCAATCTAGCAATGTAATTCAGT |
| Integrin-beta1 N363Q Rev | ACTGAATTACATTGCTAGATTGGGCAGATAATGTTCCTACT |
| pX330-mouse Mgat5 guide 1 For | caccGTACATTAAGGCACTGGCAG |
| pX330-mouse Mgat5 guide 1 Rev | aaacCTGCCAGTGCCTTAATGTAC |
| pX330-mouse Mgat5 guide 2 For | caccGCTGTCATGACACCAGCGTA |
| pX330-mouse Mgat5 guide 2 Rev | aaacTACGCTGGTGTCATGACAGC |
| Mgat5 genotyping For | CAGAATGGAAGCCCAGCAAGGAA |
| Mgat5 genotyping Rev | CTCATCCCTCCACGTCCCCTCAC |
| pX330-mouse St6gal1 guide 1 For | caccCATTCACGTGGTCTCGAAGG |
| pX330-mouse St6gal1 guide 1 Rev | aaacCCTTCGAGACCACGTGAATG |
| pX330-mouse St6gal1 guide 2 For | caccCATACTCAAAACTTAACCCC |
| pX330-mouse St6gal1 guide 2 Rev | aaacGGGGTTAAGTTTTGAGTATG |
| St6gal1 genotyping For | CAAGGTATTCCAGATGCCGAAG |
| St6gal1 genotyping Rev | CAGCCTTGGTTCTGAAGTTCTCC |
| pX330-mouse Itgb1 guide 1 For | caccGCTGGGGTTGTGCTAATATA |
| pX330-mouse Itgb1 guide 1 Rev | aaacTATATTAGCACAACCCCAGC |
| pX330-mouse Itgb1 guide 2 For | caccGTTGGTCAGCAACGCATATC |
| pX330-mouse Itgb1 guide 2 Rev | aaacGATATGCGTTGCTGACCAAC |
| Itgb1 genotyping For | GGTCTCTTCCCACTCATCTA |
| Itgb1 genotyping Rev | AACTCCCTTTCTGGCTCAGA |
| CD81 For | AAGAATTGGGATCCGAATTCATGGGAGTGGAGGGCTGC |
| Halo7 Rev | GTTGACTCTAGAGCGGCCGCTTAGCCGGAAATCTCGAGCG |
| pPBZeo For | GCGGCCGCTCTAGAGTCA |
| pPBZeo Rev | GAATTCGGATCCCAATTCTTTGCC |

**Movies S1.** Movies showing the simultaneous observation of sEV-CD63Halo7-TMR particles (green) and laminin components (magenta) on an iMEF cell. sEVs were derived from intact B16 cells.

**Movies S2.** Movies showing the simultaneous observation of sEV-CD63Halo7-TMR particles (green) and laminin components (magenta) on an iMEF cell. sEVs were derived from MGAT5 KO B16 cells.

**Movie S3.** Movies showing the simultaneous observation of sEV-CD63Halo7-TMR particles (green) and laminin components (magenta) on an iMEF cell. sEVs were derived from ST6GAL1 KO B16 cells.

**Movie S4.** Movies showing the simultaneous observation of sEV-CD63Halo7-TMR particles (green) and laminin components (magenta) on an iMEF cell. sEVs were derived from integrin β1-rescued B16 cells.

**Movie S5.** Movies showing the simultaneous observation of sEV-CD63Halo7-TMR particles (green) and laminin components (magenta) on an iMEF cell. sEVs were derived from integrin β1 N269Q-expressed B16 cells.
